# African-specific alleles modify risk for asthma at the 17q12-q21 locus in African Americans

**DOI:** 10.1186/s13073-022-01114-x

**Published:** 2022-09-29

**Authors:** Charles Washington, Matthew Dapas, Arjun Biddanda, Kevin M. Magnaye, Ivy Aneas, Britney A. Helling, Brooke Szczesny, Meher Preethi Boorgula, Margaret A. Taub, Eimear Kenny, Rasika A. Mathias, Kathleen C. Barnes, CAAPA, Monica Campbell, Camila Figueiredo, Nadia N. Hansel, Carole Ober, Christopher O. Olopade, Charles N. Rotimi, Harold Watson, Gurjit K. Khurana Hershey, Carolyn M. Kercsmar, Jessica D. Gereige, Melanie Makhija, Rebecca S. Gruchalla, Michelle A. Gill, Andrew H. Liu, Deepa Rastogi, William Busse, Peter J. Gergen, Cynthia M. Visness, Diane R. Gold, Tina Hartert, Christine C. Johnson, Robert F. Lemanske, Fernando D. Martinez, Rachel L. Miller, Dennis Ownby, Christine M. Seroogy, Anne L. Wright, Edward M. Zoratti, Leonard B. Bacharier, Meyer Kattan, George T. O’Connor, Robert A. Wood, Marcelo A. Nobrega, Matthew C. Altman, Daniel J. Jackson, James E. Gern, Christopher G. McKennan, Carole Ober

**Affiliations:** 1 Department of Human Genetics, The University of Chicago, 928 E. 58th St. CLSC 507C, Chicago, IL 60637 USA; 2 Department of Medicine, Johns Hopkins University, Baltimore, MD USA; 3 Department of Medicine, University of Colorado Denver, Aurora, CO USA; 4 Department of Biostatistics, Bloomberg School of Public Health, Johns Hopkins University, Baltimore, MD USA; 5 Department of Medicine, Icahn School of Medicine at Mount Sinai, New York, NY USA; 6 Consortium on Asthma in African-ancestry Populations in the Americas, Denver, USA; 7 Division of Asthma Research, Cincinnati Children’s Hospital, Cincinnati, OH USA; 8 Department of Medicine, Division of Pulmonary, Allergy, Sleep, and Critical Care Medicine, Boston University School of Medicine, Boston, MA USA; 9 Ann and Robert H. Lurie Children’s Hospital of Chicago, Chicago, IL USA; 10 University of Texas Southwestern Medical Center, Dallas, TX USA; 11 Children’s Hospital Colorado and University of Colorado School of Medicine, Aurora, CO USA; 12 Children’s National Hospital and George Washington University School of Medicine and Health Sciences, Washington, DC USA; 13 University of Wisconsin School of Medicine and Public Health, Madison, WI USA; 14 NIH/NIAID, Bethesda, MD USA; 15 Rho Federal Systems Division, Inc, Durham, NC USA; 16 The Channing Division of Network Medicine, Department of Medicine, Brigham and Women’s Hospital and Department of Environmental Health, Harvard T.H. Chan School of Public Health, Harvard University, Boston, MA USA; 17 Department of Medicine, Vanderbilt University School of Medicine, Nashville, TN USA; 18 Department of Public Health Sciences, Henry Ford Health Systems, Detroit, MI USA; 19 Department of Pediatrics, University of Wisconsin School of Medicine and Public Health, Madison, WI USA; 20 Asthma and Airway Disease Research Center, University of Arizona, Tucson, AZ USA; 21 Department of Medicine, Division of Clinical Immunology Icahn School of Medicine at Mount Sinai, New York, NY USA; 22 Department of Medicine, Henry Ford Health Systems, Detroit, MI USA; 23 Department of Pediatrics, Monroe Carell Jr Children’s Hospital at Vanderbilt University Medical Center, Nashville, TN USA; 24 Department of Pediatrics, Columbia University Medical Center, New York, NY USA; 25 Pulmonary Center, Boston University School of Medicine, Boston, MA USA; 26 Department of Pediatrics, Johns Hopkins University, Baltimore, MD USA; 27 Immunology Division, Benaroya Research Institute Systems, Seattle, WA USA; 28 Department of Medicine, University of Washington, Seattle, WA USA; 29 Department of Statistics, University of Pittsburgh, Pittsburgh, PA USA

**Keywords:** Asthma, Fine mapping, Integrated omics, Whole-genome sequencing, Health disparities

## Abstract

**Background:**

Asthma is the most common chronic disease in children, occurring at higher frequencies and with more severe disease in children with African ancestry.

**Methods:**

We tested for association with haplotypes at the most replicated and significant childhood-onset asthma locus at 17q12-q21 and asthma in European American and African American children. Following this, we used whole-genome sequencing data from 1060 African American and 100 European American individuals to identify novel variants on a high-risk African American–specific haplotype. We characterized these variants in silico using gene expression and ATAC-seq data from airway epithelial cells, functional annotations from ENCODE, and promoter capture (pc)Hi-C maps in airway epithelial cells. Candidate causal variants were then assessed for correlation with asthma-associated phenotypes in African American children and adults.

**Results:**

Our studies revealed nine novel African-specific common variants, enriched on a high-risk asthma haplotype, which regulated the expression of *GSDMA* in airway epithelial cells and were associated with features of severe asthma. Using ENCODE annotations, ATAC-seq, and pcHi-C, we narrowed the associations to two candidate causal variants that are associated with features of T2 low severe asthma.

**Conclusions:**

Previously unknown genetic variation at the 17q12-21 childhood-onset asthma locus contributes to asthma severity in individuals with African ancestries. We suggest that many other population-specific variants that have not been discovered in GWAS contribute to the genetic risk for asthma and other common diseases.

**Supplementary Information:**

The online version contains supplementary material available at 10.1186/s13073-022-01114-x.

## Background

Genome-wide association studies (GWAS) have identified thousands of loci associated with hundreds of common traits and diseases, providing insights into pathogenic pathways and yielding a plethora of candidate causal single-nucleotide polymorphisms (SNPs) and genes for follow-up studies. Yet the overwhelming majority of GWAS have been performed in individuals of European or Asian ancestry with poor representation of global diversity [1, 2]. Even if many causal variants are shared between populations [3], some will surely differ, and some may even be population-specific [4, 5]. The full range of risk variants will never be discovered from studies that are limited to European-ancestry populations. Thus, imprecise predictors of genetic risk in African-ancestry populations will arise from a variety of mechanisms, including a relative dearth of knowledge of African-specific risk variants.

The 17q12-q21 childhood-onset asthma locus provides an edifying example of multi-ancestry complexities of genetic risk. A distinguishing feature of this locus is that it is the most statistically significant and most replicated childhood-onset asthma locus in GWAS of European-ancestry populations [6–8], as well as in a multi-ancestry [9] and an African-admixed [10] asthma GWAS. However, whereas odds ratios (ORs) for childhood-onset asthma at the most significant SNP in white British individuals was 1.40 (95% confidence interval [CI] 1.36, 1.44) [8], ORs for pediatric asthma at the most significant SNPs were 1.25 (CI 1.20, 1.29) in the multi-ancestry GWAS [9] and 1.35 (CI 1.13, 1.35) in the African-admixed GWAS [10]. In the African-admixed GWAS, the magnitude of the effect size was inversely correlated with the proportion of African ancestry [10]. These combined data suggested that the same SNPs at the 17q12-q21 locus may have different effects on asthma risk in individuals with African ancestry.

A second characteristic of this locus is ancestry-specific patterns of linkage disequilibrium (LD) [11]. Extensive LD in European-ancestry populations results in a 150-kilobase (kb) block of tightly linked SNPs. Therefore, although the lead SNP in European-ancestry GWAS often differs between studies, it always resides on the same extended haplotype (reviewed in ref. [11]). In contrast, considerably less LD occurs at this locus on African-ancestry chromosomes and, as a result, SNPs that tag causal variant(s) in European-ancestry populations may not tag the same causal variants in African-ancestry population, possibly accounting for the smaller effect sizes observed at GWAS loci in these populations.

A final feature of the 17q12-q21 locus is that SNPs spanning the 150-kb LD block in European-ancestry populations are expression quantitative trait loci (eQTLs) for two genes, ORM1-like 3 (*ORMDL3*) and gasdermin B (*GSDMB*), in blood cells, lung tissue, and/or airway epithelial cells [6, 12–16]. The reduced LD on African-ancestry chromosomes and the eQTL effects have recently been leveraged for fine mapping, revealing that the association with childhood-onset asthma at this locus is due to genetic variation influencing the expression of *GSDMB* [17, 18], particularly in airway epithelial cells [17]. The childhood-onset asthma–associated alleles at a missense SNP in *GSDMB*, rs2305480-G, had an estimated OR for childhood-onset asthma of 1.17 (CI 1.05, 1.30) in a meta-analysis of 3904 African American subjects [17]. Using rs2305480 and four other tag SNPs across the 150-kb core region, alleles at two SNPs associated with risk for childhood-onset asthma in European-ancestry children (rs12936231, rs4065275) had estimated ORs <1 in African American children [17], suggesting that differences in the local haplotype structure may be contributing to the 17q12-q21 disease liability for childhood-onset asthma in African-ancestry populations.

Based on these earlier results, we hypothesized that additional variation on African-ancestry chromosomes within the core region of the 17q12-q21 locus modifies risk for childhood-onset asthma. In this study, we explore this hypothesis by first performing haplotype-based analyses in the same 868 African American children as in our earlier study [17] and then use whole-genome sequences from 100 European American and 1060 African American individuals to characterize the haplotype structure at this locus at single-nucleotide resolution. Ultimately, we defined a 26.3-kb critical region in the 17q12-q21 core region of a high-risk haplotype that included nine African-specific variants. These variants were eQTLs in airway epithelial cells only for gasdermin A (*GSDMA*), overlapped with Encyclopedia of DNA Elements (ENCODE) [19] enhancer annotations in multiple cell types and with open chromatin in airway epithelial cell lines, and physically interacted with the promoter of *GSDMA* by promoter capture (pc)Hi-C in primary airway epithelial cells [20]. The novel variants were also associated with measures of severity in African American children and adults. We suggest that additional examples of non-European-ancestry–specific variants may underlie ancestry-specific differences in disease liability and effect size estimates at other asthma loci as well as at loci associated with other common diseases. An overview of our study design is shown in Additional file 1: Fig. S1.

## Methods

### Study cohorts

The individual cohorts used in these studies are described in Additional file 1: Supplementary Methods.

### 17q12-q21 Haplotype Analysis in the Children’s Respiratory and Environmental Workgroup (CREW) Cohort [21]

Most of the participants in CREW were not genotyped with genome-wide SNPs. Therefore, we genotyped nine tag SNPs across the extended 17q12-q21 region using TaqMan assays in a previous study [17]. These SNPs were selected to capture the LD pattern in African-ancestry populations and to represent variants that were either (i) the lead SNP in a previous asthma GWAS, or (ii) a known eQTL for any of the genes across the extended locus, as presented in Stein et al. [11]. Based on our previous results [17], we selected the five SNPs that tagged the core region for these studies. We performed haplotype analysis in the 1647 parent-reported Non-Hispanic White (NHW) and 868 parent-reported Non-Hispanic Black (NHB) participants who were followed to at least age 6 years and were genotyped for SNPs at the 17q12-q21 locus as part of our earlier study [17]. Because this locus is associated with wheezing illnesses in early life [12, 22–24] and asthma by age 5 years [25], we used healthcare provider–diagnosed asthma by age 6 years to define cases and never-diagnosed asthma by age 6 or by the last age at which the participant was studied after age 6 to define the controls. Of the 1647 NHW subjects, 300 were asthma cases and 1347 were non-asthmatic controls; among the 868 NHB subjects, 318 were asthma cases and 550 were non-asthmatic controls. Additional details on these cohorts and genotyping methods are provided in our earlier report [17] and in Additional file 1: Supplementary Methods.

Because we did not see evidence of association between asthma and SNPs in the regions proximal and distal to the core region in NHB subjects in our previous study [17], we included here the five SNPs that were genotyped in the 17q12-q21 core region to estimate haplotypes separately in the NHW and NHB individuals. We used haplo.em, as described in Schaid et al. [26], and the default settings in the haplo.stats package for genotype data. The median estimated haplotype posterior probability for the included individuals was 1.0 (interquartile range 0.99 to 1.0; minimum 0.45). We tested the additive effects of each haplotype on asthma risk relative to the European-protective 5-SNP haplotype (rs12936231-G_rs2305480-A_rs7216389-C_rs4065275-A_rs8076131-G) separately in NHW and NHB subjects using logistic regression to generate ORs and 95% CIs, as implemented in haplo.glm [27]. This method uses the posterior probabilities of the haplotype assignments iteratively to weight the regression coefficients. Only haplotypes with frequency ≥0.05 were included, resulting in one test (two haplotypes) in the European American sample and three tests (four haplotypes) in the African American sample. Sex and recruitment city were included as covariates in each model. Because genome-wide genotypes were not available for all the subjects in this study, we could not correct for genetic ancestry in this analysis.

### Whole-genome sequence curation and phasing

We included publicly available whole-genome sequence data from the EVE [28] and Consortium on Asthma in African-ancestry Populations in the Americas (CAAPA) [29, 30] studies, as well as newly generated whole-genome sequences [31] in the Asthma Phenotypes in the Inner City (APIC) [32] and Urban Environment and Childhood Asthma (URECA) [33] cohorts. These cohorts are further described in Additional file 1: Supplementary Methods.

We first pruned the files to include African American individuals in EVE (*n*=90), CAAPA (*n*=320), APIC (*n*=321), and URECA (*n*=329), and European American individuals in EVE (*n*=100). African American or European American ancestries were determined by ancestry principal component (PC) analysis in each cohort. All EVE and APIC subjects had doctor-diagnosed asthma; CAAPA included 168 subjects with doctor-diagnosed asthma, and URECA included 176 subjects with doctor-diagnosed asthma. These datasets are described in Table 1 and in Additional file 1: Supplementary Methods. We used the Michigan Imputation Server [34] to phase all variants on chromosome 17, separately in the European American and African American sequences, using the Haplotype Reference Consortium^4^ (r1.1 2016) as the reference panel. Standard quality control checks were used [34], including removal of duplicated sites, non-SNP variants, monomorphic sites, invalid alleles, and SNPs with call rates less than 90%.

**Table 1:** Whole-genome sequence datasets

Study	Race/ethnicity	Sample sizes			dbGaP accession number	
		Total sample	Asthma cases	Cases homozygous for a 5-SNP haplotype		Reference
URECA	African American	329	176	36	phs002921.v1.p1	[33]
APIC	African American	321	321	87	phs002921.v1.p1	[32]
CAAPA	African American	320	168	40	phs001123.v2.p1	[30]
EVE	African American	90	90	14	phs001156.v2.pa	[28]
EVE	European American	100	100	44	phs001156.v2.p1	[28]

*URECA* Urban Environment and Childhood Asthma, *APIC* Asthma Phenotypes in Inner-City Children, *CAAPA* Consortium on Asthma among African-Ancestry Populations in the Americas

See “Methods” for descriptions of each cohort

### Visualizing haplotypes

**Fig. 1:**
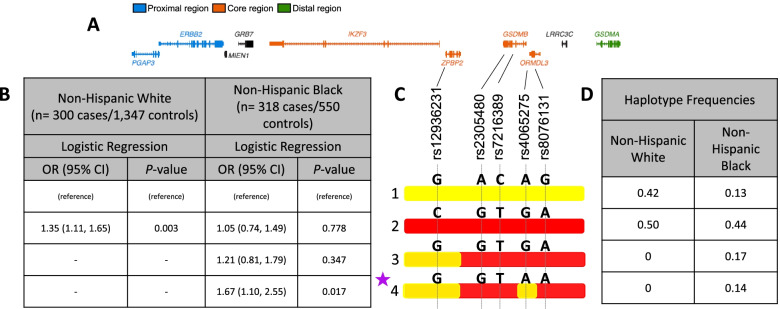
Common haplotypes at the 17q12-q21 locus. **A** Ten genes and their relative locations at the extended locus (chr17:37815888-38143768; build hg19). Genes at the core, proximal, and distal regions are color coded (modified from Stein et al. [11] and Ober et al. [17]). The five variants defining the core region haplotypes and their locations relative to each gene are shown. **B** Results of logistic regression analysis for haplotypes with frequencies ≥0.05; haplotype 1 is the reference haplotype. **C** Allelic composition of the five variants on four haplotypes. Yellow background corresponds to alleles on the non-risk haplotype (haplotype 1) and red background corresponds to alleles on the risk haplotype (haplotype 2 in European-ancestry populations). The purple star denotes the high-risk haplotype in African Americans. **D** Haplotype frequencies

To visualize haplotypes, we used ChromoPainter [35], a tool used for defining haplotypes in sequence data. For this step, we used phased chromosome 17 sequences from all individuals with whole-genome sequences. We then selected the individuals who were homozygous for the 5 SNPs that defined the core region haplotype and tagged the European risk and protective haplotypes identified in the haplotype studies described above and in Fig. 1. We focused on homozygous individuals to maximize the accuracy of the phasing. ChromoPainter requires two file types: a phased genotype file and a genetic map designating recombination distances between SNPs. For the first, we converted the phased .vcf genotypes files for all chromosome 17 variants into the .phase format. For “painting” the sequences, we used the 1000Genomes recombination map [36] for the European American chromosomes and an African American–specific map for the African American chromosomes [37]. ChromoPainter was run separately for each population across chromosome 17.

Using these two files, ChromoPainter “paints” each phased chromosome by comparing it to all other phased chromosomes in the sample. For each chromosome at each SNP, the expected probability that an index chromosome has been copied from each of the other chromosomes is determined using a Hidden Markov Model. ChromoPainter then outputs a matrix for each chromosome with the number of rows equal to the number of SNPs and the number of columns equal to the number of phased chromosomes. Each cell within this matrix contains the probability that the column’s chromosome was copied by the index chromosome at that SNP based on the sequence similarity of the two chromosomes and the rate of recombination between the current and previous SNP (Additional file 1: Fig. S2).

**Fig. 2:**
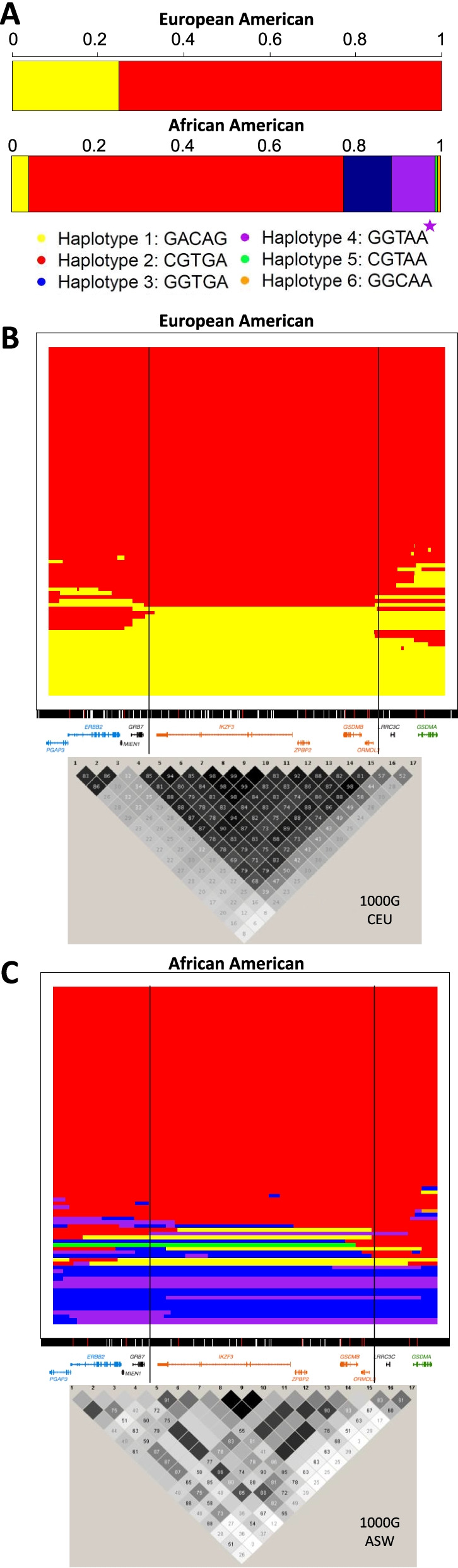
17q12-q21 haplotypes in whole-genome sequences from individuals with asthma and homozygous for the 5-SNP haplotype. **A** Bar graph of haplotypes present in 44 Europeans (88 chromosomes) and 177 African Americans (354 chromosomes). The allelic composition and color code of each 5-SNP haplotype are shown under the bars. The purple star designates the African American high-risk haplotype 4. **B** ChromoPainter display of the haplotypes (88 chromosomes) in European Americans. **C** ChromoPainter display of 89 randomly selected chromosomes from the 354 chromosomes in African Americans. All chromosomes are shown in Additional file 1: Fig. S3. The LD plots include 17 SNPs previously associated with asthma in GWAS or with expression of genes at this extended locus, as previously described [11], are shown below the ChromoPainter displays. LD data are from 1000 Genomes (CEU and ASW, respectively); *r*^2^ values are shown in each diamond. The darker the diamond, the more LD; black diamonds are *r*^2^ = 1.0

Chromosomes corresponding to the highest posterior probability for each SNP across the chromosome were selected, and we then assigned haplotypes (different colors) for each row of phased SNPs based on the 5-SNP haplotype with the highest probability (see Fig. 2). Each horizontal line represents a single chromosome, with coloration based on the highest-probability donor haplotype at each locus on the chromosome. Chromosomes change color as the highest-probability donor haplotype changes according to the maximum posterior probability at each base pair. The median of the maximum posterior probabilities across all base pairs (not specifically at haplotype switches) for each chromosome varied from 0.122 to 1.0 (median = 0.883) in African Americans and 0.246 to 1.0 (median = 0.824) in European Americans. This process was repeated for each chromosome in our sample (2n=88 European American and 354 African American chromosomes from individuals homozygous for the 5-SNP haplotype). After running ChromoPainter for chromosome 17, we focused on the 17q12-q21 region corresponding to chr17:39674647-39972395 (hg38), which included the proximal, core, and distal regions, as defined by Stein et al. [11]. LD plots of selected SNPs at this locus were generated using Haploview [38] with data from 1000 Genomes CEU and ASW populations to represent European American and African American populations, respectively.

### Identifying novel variants on the high-risk haplotype

The ChromoPainter analysis allowed us to delineate a critical region that was shared among individuals with the high-risk 5-SNP haplotype. Variants in the critical region that were unique to the high-risk haplotype in the African American sequences were selected by first creating consensus sequences of the critical region in individuals who were homozygous for one of the six haplotypes (see Additional file 1: Supplementary Methods for additional details).

The African American–specific high-risk consensus sequence was compared to each of the other five consensus sequences to identify SNPs that occurred only on the African American–specific high-risk haplotype. Candidate causal variants were identified at base pair positions at which the allele on the African American high-risk consensus haplotype always differed from the allele present on the other five consensus haplotypes among the 5-SNP haplotype homozygous individuals.

### eQTL mapping of African-specific variants on the high-risk haplotype

eQTLs from RNA-seq data collected from upper airway (nasal) cells collected at age 11 were available for 189 African American children in the URECA cohort [39]. See Additional file 1: Supplementary Methods for a description of the cohort. For eQTL studies, we included all SNPs within the 26.3-kb critical region with minor allele frequencies ≥0.05 and used an additive effects linear model, including sex, recruitment site, sequencing batch, epithelial cell proportion, ancestry PC1-3, and 11 latent factors as covariates, as implemented in Matrix eQTL [40] for a region-wide analysis*.* Latent factors were included to adjust for unwanted variation [41]. We included all African-specific variants in the 26.3-kb critical region and tested for genotype effects of each variant on the expression of all genes whose TSS were within ±500 kb (*n*=27 genes). We used an FDR threshold of ≤0.10 to control the false-positive rate. As local references, we also performed post hoc analyses of eQTLs for the asthma-associated rs2305480 SNP, a known eQTL for *GSDMB*, and for SNPs across the *GSDMA* gene that were previously reported eQTLs for *GSDMA*.

To replicate our findings, we performed eQTL studies using nasal epithelial cell gene expression from RNA-seq data and genotypes (MEGA array; Illumina) for 534 participants in CAAPA2 (unpublished) (see Additional file 1: Supplementary Methods for a description of the CAAPA cohort). Genotypes for one of the African-specific variants (rs28623237) on the high-risk haplotype 4 were available for these individuals. We tested for association between rs28623237 genotype and *GSDMA* expression (cpm) using a linear model that included age, sex, recruitment site, RIN, GC content, library batch, and the first two ancestry PCs as covariates.

### Annotating variants for regulatory function

Annotation of regulatory elements in the region containing the variants specific to the African American high-risk haplotype was conducted using publicly available data for regulatory regions in ENCODE [19]. We specifically examined Chromatin Immunoprecipitation-seq data for H3K27ac, a marker of active enhancers, and DNase clusters, marks of open chromatin. Finally, to identify putative enhancers that overlap with the African-specific variants, we used publicly available pcHi-C data in primary bronchial epithelial cells (GSE 152549) [20]. We considered only Hi-C loops with CHiCAGO scores ≥5 [42].

### Testing associations between African-specific variants and clinical phenotypes

To examine the phenotypic effects of the African-specific variants, we used harmonized phenotype data in the APIC and URECA cohorts for seven asthma-associated quantitative traits in African American individuals: pre-bronchodilator % predicted FEV1 (*n*=607), FEV1/FVC (*n*=596), FeNO (*n*=423), bronchodilator response (*n*=589), total serum IgE (*n*=604), blood eosinophil count (*n*=606), and blood neutrophil count (*n*=606). These cohorts are described in Additional file 1: Supplementary Methods and Table S1. We used a linear mixed model [43] to test for additive effects of the African-specific variant, including age, sex, asthma status, and ancestry PC1-3 as covariates and a kinship matrix as a random effect to adjust for relatedness. To adjust for seven tests, we tested the joint null hypothesis that none of the associations are significant [44]. This test is an alternative to the more commonly used, but highly conservative, Bonferroni adjustment. We adjusted the null distribution to account for the correlations between the seven phenotypes.

We also tested for associations with asthma severity in 63 African American adults who participated in the Chicago Asthma Genetics (CAG) study [45, 46] (see Additional file 1: Supplementary Methods). Adult asthmatics were assigned as mild (*n*=19), moderate (*n*=18), or severe (*n*=26) using STEP classifications [47], which are based on both steroid use and lung function measures. Because whole-genome sequences, and therefore genotypes for the African-specific SNPs, were not available for these individuals, we imputed the 5-SNP haplotype from phased genotype data (Michigan Imputation Server [34]), and then tested for associations between of the high-risk haplotype 4 (G-G-T-A-A) frequencies and STEP classification using ordinal logistic regression, including age, sex, current smoking status, and ancestry PC1-3 as covariates. Association between asthma severity and the combined effects of rs2305480 genotype and carriage of the high-risk haplotype 4 was tested using ordinal logistic regression, including the same covariates as above.

## Results

### 17q12-q21 haplotype associations with asthma in European American and African American individuals

We first examined associations between the common haplotypes (frequency ≥0.05) and asthma in the same parent-reported NHW and NHB subjects in the Children’s Respiratory and Environment Workgroup (CREW) cohorts included in our previous study [17]. Haplotypes were assigned based on five SNPs that tagged the core region of the 17q12-q21 locus. The SNPs included the missense SNP, rs2305480, in *GSDMB*, previously reported to be the lead SNP in these subjects [17] and in near perfect LD with a *GSDMB* splice variant, rs11078928, in both European-ancestry and African-ancestry populations [18]. The two common 5-SNP haplotypes in these samples, including the non-risk (haplotype 1) and risk (haplotype 2) haplotypes, accounted for 92% of haplotypes in NHW individuals and 57% of the haplotypes in the NHB individuals, with the same directions of effect (Fig. 1). Two other haplotypes were common in the NHB (frequencies 0.17 and 0.14 for haplotypes 3 and 4, respectively) but absent in the NHW (Fig. 1). An additional 13 haplotypes in the NHW and 11 in the NHB were present at frequencies less than 0.05 (Additional file 1: Table S2).

Consistent with our earlier study of individual SNPs [17], the haplotype carrying the rs2305480-G allele (haplotype 2) was associated with asthma in the NHW individuals (OR = 1.35 [CI 95% 1.11, 1.65]; *p* = 0.0025). In the NHB individuals, a different haplotype also carrying the rs2305480-G allele (haplotype 4) was most strongly associated with asthma (OR = 1.67 [CI 95% 1.10, 2.55]; *p* = 0.017), whereas two other haplotypes carrying the rs2305480-G allele (haplotypes 2 and 3) had estimated ORs greater than 1.0 but were not statistically significant in this sample. These results suggested that haplotype 4 carries additional variation that increases risk for asthma in NHB individuals.

To identify variants on haplotype 4 that may be contributing to asthma risk, we first characterized the variation at the 17q12-q21 locus using whole-genome sequences from four datasets including either asthma cases only (APIC and EVE) or both asthma cases and controls (URECA and CAAPA) (Table 1). To maximize phasing accuracy, we first focused on sequences from asthma cases who were homozygous for the 5 common haplotype-defining SNPs (44 European Americans and 177 African Americans). Only haplotypes 1 and 2 were observed in the homozygous sequences of the European American asthma cases (Fig. 2A). However, in the African American asthma cases who were homozygous for the 5 haplotype-defining SNPs, the four common haplotypes (haplotypes 1, 2, 3, 4) and two others (haplotypes 5 and 6) were observed. Haplotypes 5 and 6 both carried the asthma-associated rs2305480-G allele and differed from the African American high-risk haplotype 4 by one or two of the other haplotype-defining SNPs, respectively (Fig. 2A).

To gain an initial overview of the sequence structure across the extended 17q12-q21 locus, we visualized haplotypes extending beyond the core region to include the proximal and distal regions [11] in each population using ChromoPainter [35] (see “Methods”). Nearly all haplotype “switching,” which represents historical recombination events, in the 88 European American sequences were at the boundaries of the core region or within the proximal and distal regions (Fig. 2B), consistent with LD patterns in a European-ancestry reference population (CEU). These sequences revealed greater diversity and more historical recombination in African Americans, including numerous switches within the core region, also consistent with LD patterns in an African American reference population (ASW) (Fig. 2C, Additional file 1: Fig. S3). Haplotype frequencies in each whole-genome sequence dataset are shown in Additional file 1: Table S3.

### Defining a critical region and African-specific risk variants on haplotype 4

Because variants in the proximal and distal regions were not associated with asthma in African Americans in our previous study [17] and because of the observed haplotype structures and LD patterns in African Americans (Fig. 2C), we focused on the sequences in asthma cases homozygous for the high-risk haplotype 4 (defined by 5 SNPs) to first identify the chromosomal region(s) shared by all haplotype 4 homozygotes (2N=36). Examining the ChromoPainter displays revealed a 23.9-kb region that was shared by haplotype 4 sequences and bounded by at least two recombination events on either side, providing more confidence in the boundaries (Additional file 1: Fig. S4). We then extended the region 1.2 kb (5%) on either side to capture any additional variants that may be excluded based on the small number of observed recombination events. Ultimately, we examined a 26.3-kb region that extended from intron 6 in *GSDMB* to 6.1 kb upstream of the *ORMDL3* transcription start site (TSS). We refer to this 26.3-kb segment as the “critical region.”

To identify variants that were present in the critical region of haplotype 4 but not on any of the other homozygous haplotypes, we defined consensus sequences of this region for the European American haplotypes 1 and 2 and the African American haplotypes 1 through 4 (see “Methods”). We then conducted pairwise comparisons between sequence variants in the high-risk haplotype 4 critical region and each of the five other haplotypes. Among the 58 variant sites in the critical region, nine were specific to the haplotype 4 consensus sequence, occurring at frequencies between 0.75 and 0.97 in African Americans who were homozygous for haplotype 4 (Table 2). We then expanded the sample to include all sequences from cases and controls and not just those homozygous for the 5-SNP haplotypes. The nine variants were absent in 187 European American sequences, were highly enriched in 386 African American haplotype 4 sequences (frequencies between 0.495 and 0.756), and present in lower frequency on the other 1511 African American haplotypes (frequencies between 0.019 and 0.267) (Table 2). These frequency distributions are similar to those observed in worldwide populations (Additional file 1: Table S4). These data suggested that one or more of these nine variants contribute to asthma risk in African Americans.

**Table 2:** Location and frequencies of the nine novel African-specific SNPs in the full sample

rsID	Variant	Position (hg38)	Location	EA_Hap1 (2n=69)	EA_Hap2 (2n=118)	AA_Hap1 (2n=212)	AA_Hap2 (2n=891)	AA_Hap3 (2n=408)	AA_Hap4 (2n=386)	AA_Hap4 Asthmatic, Homozygotes (2n=36)
rs150276395	G->A	chr17:39908449	*GSDMB* intron 6	0	0	0.024	0.039	0.042	0.53	0.78
rs8065520	T->C	chr17:39915395	*GSDMB* intron 2	0	0	0.066	0.094	0.270	0.79	0.97
rs73985226	A->G	chr17:39920081	Intergenic	0	0	0.024	0.037	0.029	0.50	0.78
rs73985227	G->A	chr17:39921437	*ORMDL3* 3’UTR	0	0	0.024	0.038	0.032	0.52	0.78
rs28623237	T->C	chr17:39924694	*ORMDL3* intron 3	0	0	0.033	0.062	0.037	0.76	0.94
rs73985229	G->A	chr17:39925400	*ORMDL3* intron 1	0	0	0.019	0.039	0.029	0.53	0.78
rs113282230	A->T	chr17:39927157	*ORMDL3* intron 1	0	0	0.019	0.039	0.029	0.53	0.78
rs113571956	A->T	chr17:39927234	*ORMDL3* intron 1	0	0	0.019	0.039	0.029	0.53	0.78
rs73985230	T->C	chr17:39929476	Intergenic	0	0	0.019	0.039	0.027	0.50	0.75

The asthmatics who were homozygous for the high-risk haplotype 4 are shown in the last column. Only haplotypes included in the ChromoPainter analyses (Fig. 1) are shown. Locations are based on GENCODE (V36) gene track data

*2n* number of chromosomes, *EA* European American, *AA* African American

### Functionally characterizing the African American–specific variants on haplotype 4

**Fig. 3:**
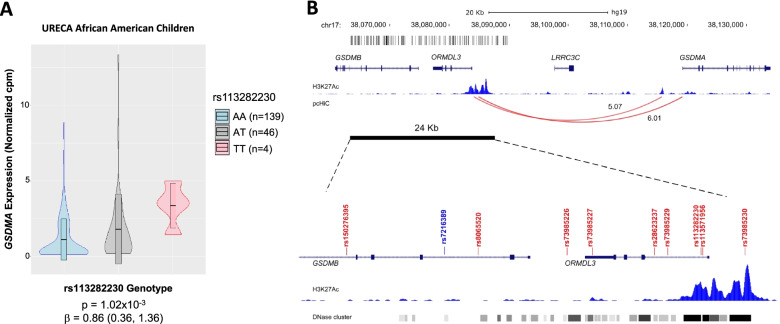
Functional characteristics of the African-specific novel variants on the high-risk asthma haplotype. **A** rs113282230, as a representative of the novel variants, is an eQTL for *GSDMA* but no other genes in upper airway epithelial cells (see Table 3 for results with all nine variants and Additional file 2 for results with all genes). **B***Upper panel*: Chromosomal region from the 26.3-kb critical region (thick black bar) to the *GSDMA* gene on chromosome 17q12-q21. Vertical lines at the top show the locations of all variants in the critical region. The location of the four genes in the region, showing pcHi-C interactions (red arc) from a region in intron 1 of *ORMDL3* to *GSDMA*. H3K27ac peaks (read counts; light blue tracks) in primary normal human epidermal keratinocytes (NHEK) (ENCODE) are shown in a region overlapping with the pcHi-C capture. *Lower panel*: Close-up of the 26.3-kb critical region. The nine African-specific variants enriched on haplotype 4 and eQTLs for *GSDMA* are shown in red. The same HEK27ac peaks as in upper panel, in addition to tracks of DNase clusters across all ENCODE cell lines, are shown. The darker the tracks the denser the DNase cluster. Two of the nine variants, rs113282230 and rs113571956, overlap with the marks of an active enhancer (H3K27ac), open chromatin (DNAse), and a putative enhancer (pcHi-C). See Fig. 4 and Additional file 1: Table S7 for additional annotations in airway epithelial cells and Additional file 1: Fig. S13 and Table S8 for additional annotations in immune cells

The nine African-specific variants that were enriched on haplotype 4 spanned from intron 6 of *GSDMB* to an intergenic region between *ORMDL3* and *LRRC3C*. We hypothesized that these variants modulate asthma risk by impacting the expression of *cis* genes. To test this hypothesis, we extracted processed RNA-seq data [39] for the 27 genes whose TSS were within 500 kb of each of the nine variants and detected as expressed in upper airway (nasal) epithelial cells from 189 African American URECA children. Ancestry principal components (PCs) 1 and 2 for URECA children are shown in Additional file 1: Fig. S5. We then performed eQTL analyses of these genes and the nine African-specific variants. At a nominal (uncorrected) *p*-value <0.05, all nine novel variants were *cis-*eQTLs for only one of the 27 genes, gasdermin A (*GSDMA*). At a false discovery rate (FDR) of ≤0.10, seven of the nine variants remained significant (*p* ≤ 2.5×10^−3^; Table 3). The results for all analyses are shown in Additional file 2. The alleles on high-risk haplotype 4 were associated with increased expression of *GSDMA* (e.g., rs113282230-T *p* = 1.02×10^−3^, *b* = 0.086; Fig. 3A). This eQTL effect on *GSDMA* expression was replicated in nasal epithelial cell transcripts from 534 individuals of African ancestry in the CAAPA2 cohort (rs28623237-G *p* = 8.65×10^−5^, *b* = 0.116; Additional file 1: Fig. S6).

**Table 3:** cis-eQTL mapping results for the nine novel variants

SNP-effect allele	Position (hg38)	Gene	***t***-statistic	Beta	***P***-value	FDR
rs150276395-G	chr17:39908449	GSDMA	2.59	0.66	0.0100	0.320
rs8065520-T	chr17:39915395	GSDMA	3.40	0.72	0.00084	0.056
rs73985226-A	chr17:39920081	GSDMA	3.22	0.83	0.0016	0.079
rs73985227-A	chr17:39921437	GSDMA	3.51	0.89	0.00059	0.055
rs28623237-C	chr17:39924694	GSDMA	3.45	0.74	0.00072	0.056
rs73985229-A	chr17:39925400	GSDMA	3.35	0.86	0.0010	0.057
rs113282230-T	chr17:39927157	GSDMA	3.35	0.86	0.0010	0.057
rs113571956-T	chr17:39927234	GSDMA	3.35	0.86	0.0010	0.057
rs73985230-C	chr17:39929476	GSDMA	3.06	0.79	0.0026	0.110

Each variant was tested for association with expression of all genes whose transcription start site is ±500 kb away and detected as expressed in airway epithelial cells from 189 African American children in the URECA cohort

Results with uncorrected *p*-values <0.05 are shown. All results are shown in Additional file 2. The *t*-statistic from Matrix eQTL is shown

Although SNPs within *GSDMA* at the distal end of the locus were more significant eQTLs for *GSDMA* in the airway epithelial cells (Additional file 1: Table S5), the LD between the six most significant African-specific SNPs in the 17q12-q21 core region and the eQTL SNPs in the *GSDMA* gene was small (LD *r*^2^ ≤ 0.28; Additional file 1: Fig. S7). Consistent with this observation, the eQTL effect of rs113282230-T was only modestly reduced when we included eQTL tag SNPs from each LD block in *GSDMA* as a covariate in the eQTL model for rs113282230 (*p* = 2.44×10^−3^, *b* = 0.744 conditioned on rs3859129 and *p*=4.69×10^−3^, *b* = 0.708 conditioned on rs4795406; see Additional file 1: Fig. S8). These conditional analyses indicate that the observed eQTL effects of the novel 17q12-q21 SNPs are independent of the eQTL SNPs in the *GSDMA* gene.

The novel variants with eQTL effects are enriched on haplotypes that also carry the main risk allele, rs2305480-G (Table 2), which is an eQTL for *GSDMB* [17]. To determine whether the effects of the novel variants on *GSDMA* expression were independent of the effects of rs2305480 on *GSDMB* expression and that each were specific eQTLs for different members of the gasdermin gene family, we performed eQTL studies with rs2305480 on *GSDMA* expression and with rs113282230 (as a surrogate for the nine novel variants) on *GSDMB* expression in the airway epithelial cells from URECA African American children (Additional file 1: Fig. S9). These results indicated that rs2305480 is an eQTL for *GSDMB* but not for *GSDMA* and rs113282230 is an eQTL for *GSDMA* but not *GSDMB*, consistent with the LD pattern between rs113282230 and other common variants in the 17q12-q21 core region (*r*^2^ < 0.11; Additional file 1: Fig. S10).

**Fig. 4:**
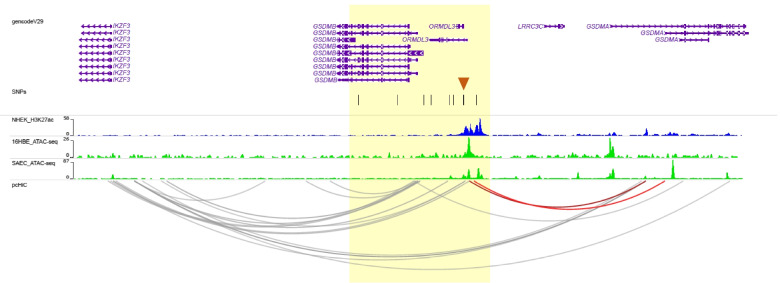
pcHi-C loops and ATAC-seq peaks at 17q12-q21 locus from *IKZF3* to *GSDMA*. The region harboring the 9 novel variants is shown in yellow and the location of the variants are show as vertical lines under the genes. The two candidate variants are indicated by an orange arrow. H3K27ac marks in NHEK (skin) cells from ENCODE are shown as blue tracks (also see Fig. 3B and Additional file 1: Fig. S11). ATAC-seq tracks of open chromatin for two airway epithelial cell lines (16HBE and SAEC) are shown in green. All pcHi-C interactions within this view in airway epithelial cells are shown. Two interactions between *GSDMA* with three of the nine variants (±1kb) were observed (shown as red loops). Two of those variants (orange arrow) were also eQTLs for *GSDMA*. All genes showing pcHi-C interactions with the 9 variants (±1kb) are shown in Additional file 1: Table S7

Because of the strong LD between the nine novel variants, it was not possible to statistically determine which variants impart functional effects on gene regulation at this locus. Therefore, we examined an active enhancer mark (H3K27ac) and areas of open chromatin assessed by DNAse in multiple cell lines from ENCODE [19] and by ATAC-seq in two airway epithelial cells lines (human bronchial epithelial cells, 16HBE, and small airway epithelial cells, SAEC). Two of the *GSDMA* eQTL variants, rs113282230 and rs113571956, overlapped with active enhancer marks, DNAse clusters in multiple cell types, and ATAC-seq peaks in airway epithelial cells (Figs. 3B and 4). DNase hypersensitivity sites of open chromatin in all ENCODE cells and in immune cells are shown in Additional file 1: Table S6 and Fig. S11, respectively. Next, we extracted published data on promoter capture Hi-C (pcHi-C) in lower airway (bronchial) epithelial cells [20] and examined interactions with the region containing the novel variants*.* Two interactions were observed between the promoter of *GSDMA* and the genomic region characterized by marks of active enhancers and open chromatin, which included rs113282230 and rs113571956 (Capture HiC Analysis of Genomic Organization [CHiCAGO] scores = 6.01 and 5.07 (Fig. 3B). Additional interactions and open chromatin marks are shown in Fig. 4 and Additional file 1: Table S7). These data suggests that rs113282230 and rs113571956 reside in an enhancer region that regulates the expression of *GSDMA* via chromatin looping and direct interaction with its promoter and provides a mechanistic explanation for how two novel variants in an intron of *ORMDL3* regulate the expression of *GSDMA*, 33.5–54.5 kb away.

To evaluate functional evidence for the 9 variants in immune cells, we used published eQTL data in the eQTL browser (https://fivex.sph.umich.edu/variant/eqtl/17_39927157?group_by=symbol&n_labels=5&study%5B%5D=Schmiedel_2018&tss_distance=500000&y_field=log_pvalue) and pcHi-C data in the Open Target browser (https://genetics.opentargets.org/variant/17_39927157_A_T). The three most significant eQTLs in immune cells were with increased expression of long non-coding RNAs *AC08112.1* and *AC090884.2* in naïve Tregs (*p*=0.0038) and Th1-17 memory cells (*p*=0.0043), respectively, and with decreased expression of *RARA* in Th1-17 memory cells (*p*=0.010) (Additional file 1: Fig. S12). None of the promoters of these eGenes interacted with regions that overlapped with the novel variants in pcHi-C data in immune cells (Additional file 1: Fig. S13 and Table S8). Thus, these combined data do not support a role for the novel variants regulating the expression of genes in immune cells.

### Associations between African-specific variants and clinical correlates of asthma

The results described above highlighted two novel African-specific variants, rs113282230 and rs113571956, that were enriched on the asthma high-risk haplotype, were eQTLs for *GSDMA*, and mapped within a putative enhancer element that physically interacted with the promoter of *GSDMA*. These SNPs were in perfect LD in our sample (*r*^2^ = 1; Additional file 1: Fig. S7), so we arbitrarily selected one (rs113282230) for further analyses with clinical measures. We first examined seven asthma-associated quantitative traits that were available for the African American children in both the APIC and URECA cohorts (*n*=607). Descriptions of these cohorts are shown in Additional file 1: Table S1; ancestry PCs 1–2 in each population are shown in Additional file 1: Fig. S5. These seven traits represented the lung function (pre-bronchodilator %predicted forced expiratory capacity at 1 s [FEV1], *n*=607; FEV1/forced vital capacity [FVC], *n*=601; bronchodilator response, *n*=588), airway inflammation (fractional exhaled nitric oxide [FeNO], *n*=423), allergic (total immunoglobulin E [IgE], *n*=604), and immune cell (blood eosinophil count and blood neutrophil count, *n*=606) components of asthma.

**Fig. 5:**
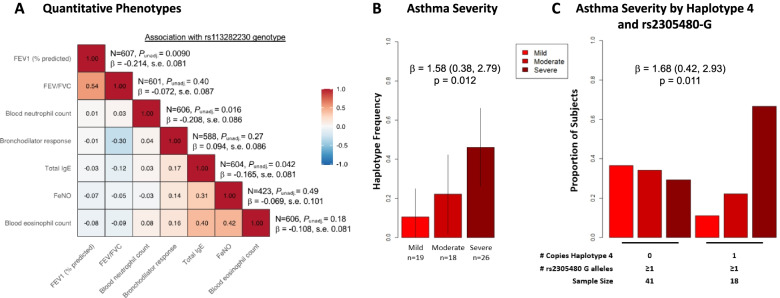
Clinical phenotype associations with the novel variants and haplotype 4. **A** Correlation plot of the seven asthma-associated quantitative phenotypes in African American children from the URECA and APIC cohorts and their association with rs113282230 genotypes. **B** Bar plot showing the frequency of the 5-SNP high-risk haplotype 4 by STEP classification categories (mild, moderate, and severe) [47] in African American adults from Chicago. Severity categories and sample sizes are shown on the *x*-axis and the frequency of haplotype 4 is shown on the *y*-axis. Haplotype 4 was used as a surrogate for the nine novel SNPs because neither whole-genome sequences nor imputed genotypes for these variants were available for these individuals. **C** Bar plot of the frequencies of asthma severity categories in African American adults with asthma who carry at least one rs2305480-G allele, stratified by the presence or absence of haplotype 4 (*x*-axis). None of these individuals were homozygous for haplotype 4

Three phenotypes were associated with rs113282230 at nominal significance (*p*<0.05): %predicted FEV1 (*p* = 9.06×10^−3^), blood neutrophil count (*p* = 0.016), and total IgE (*p* = 0.042) (Fig. 5A). The asthma risk alleles were associated with lower values of FEV1, total IgE, and neutrophil counts. None of the tests were significant after adjusting for seven tests using the conservative Bonferroni correction (*p* <0.007). However, using the correlation between *z*-scores of the seven traits, we calculated the probability of observing at least three tests with *p*<0.05 by chance and rejected the global null hypothesis that none of the traits are associated with rs113282230 (*p* = 0.0089) [44]. The results for all nine variants and all seven traits are shown in Additional file 1: Table S9.

Taken together with the chromatin annotations and the results of the haplotype studies in the CREW cohorts, these clinical data suggested that the rs113282230-T allele increases the risk of asthma in carriers of the rs2305480-G allele. To examine this more directly, we tested the additive effects of the rs113282230-T allele in rs2305480-AA (low risk) and rs2305480-GG (high-risk) homozygotes (coded as 0 or 1). If the rs113282230-T allele had no effect on risk, then rs2305480-GG homozygotes should have similar risk regardless of the number of rs113282230-T alleles. In contrast to this null expectation, we observed an increasing prevalence of asthma with increasing numbers of rs113282230-T alleles among rs2305480-GG homozygotes, although this effect did not reach significance in this sample (OR = 1.34, 95% CI 0.95, 1.88; *P* = 0.096) (Additional file 1: Fig. S14).

### Associations between African-specific variants and asthma severity

To further generalize these results to asthma severity and to adults, we examined available data on severity for 63 African American asthmatic adults who have participated in genetic studies in Chicago [45]. Because we did not have sequence data for these individuals, we used as a surrogate the 5-SNP haplotype and tested for an association between the high-risk haplotype 4 and asthma severity, defined as mild, moderate, and severe based on lung function and steroid use [47]. Consistent with the analysis of APIC and URECA children described above, the frequency of haplotype 4 increased with increasing asthma severity in African American adults (ordinal logistic regression *β*=1.58, 95% CI 0.38, 2.79; *p* = 0.012) (Fig. 5B). To directly test whether the presence of haplotype 4 adds to the risk conferred by the rs2305480-G allele, we further stratified the 59 adults who carried at least 1 copy of rs2305480-G into two groups based on whether they also carried 0 (*n*=41) or 1 (*n*=18) copies of haplotype 4 (none of the subjects carried two copies of haplotype 4). We compared the number of subjects who were classified as mild, moderate, or severe within the two groups. If haplotype 4 did not impact asthma severity beyond the effects of the rs2305480-G allele, the distributions by severity should be similar in the two groups. However, among adult asthmatics with at least one copy of the rs2305480-G allele, there was a greater proportion of severe asthma cases among those also carrying haplotype 4 compared to those not carrying haplotype 4 (ordinal logistic regression *β* = 1.68, 95% CI 0.42, 2.93; *p* = 0.011; Fig. 5C, Additional file 1: Table S10). These combined results support a role for the novel variants, which are enriched on haplotype 4, in asthma severity in both children and adults.

## Discussion

Compared to children of European ancestry, African American children have a higher prevalence of asthma [48] that develops at a younger age [49, 50] and results in lower lung function [51], poorer response to asthma therapies [52], and more emergency room visits and hospitalization for asthma [53]. Although social and behavioral inequities clearly impact disparities in health outcomes among these populations [54], genetic differences may contribute as well. That is, among the many genetic variants whose frequencies differ among worldwide populations, some may contribute to observed disparities [55]. However, the focus of large GWAS in individuals primarily of European ancestry has limited our ability to identify variants that are relevant to non-European populations [1, 2, 4]. In this study, we addressed this gap by using whole-genome sequences to identify variants at the 17q12-q21 childhood-onset asthma locus that contribute to asthma risk specifically in African American individuals. Our conclusions are based on multiple independent lines of evidence from complementary data sources, which increase the confidence of our findings [56] and underscore the importance of allelic heterogeneity at this important locus, with different variants regulating the expression of different genes. Overall, these results raise the possibility that genotype-specific risks may be modified by genetic background.

Based on previous observations of reduced effects of 17q12-q21 GWAS variants on asthma risk in African American children [9, 10, 17] and the significantly reduced LD in this region in African-ancestry populations (reviewed in ref. [11]), we hypothesized that additional variation on African-ancestry chromosomes at this locus modifies risk for childhood asthma. Analyses of the haplotype structures at single-nucleotide resolution in European American and African American individuals identified a 26.3-kb critical region on a high-risk haplotype and led to the discovery of nine common variants that are rare on European-ancestry haplotypes. Our results suggested that the novel variants are associated with increased expression of *GSDMA* via long-range chromatin interactions and are associated with a type 2 (T2) low asthma phenotype in African American children and asthma severity in African American adults.

The gasdermin family of proteins mediate pyroptosis, a form of programmed necrotic cell death initiated in response to intracellular pathogens that leads to activation of caspase-1 or caspase-4/5 and results in gasdermin-mediated pore formation in cell membranes and eventual rupture and release of pro-inflammatory cytokines such as IL-1β [57]. Expression and function of gasdermin B, encoded by *GSDMB*, has been directly linked to genetic variation at the core region of the 17q12-q21 locus and to processes relevant to asthma [58]. Two SNPs that are in near perfect LD, rs2305480 (Pro298Ser) and rs11078928 (c.662T→C), alter protein conformation and surface charge [59] and expression of the full-length transcript [18, 60], respectively. We and others have recently shown that risk for asthma conferred by variation at the 17q12-q21 locus was due to these SNPs, implicating *GSDMB* function and/or expression in airway epithelial cells in asthma pathogenesis [17, 18, 61].

Another gene in the gasdermin family, *GSDMA*, resides outside of the core region at the proximal end of the 17q12-q21 locus, where LD (*r*^2^) between the lead GWAS SNPs in the core region (i.e., rs2305480 or rs11078928) with *GSDMA* SNPs is approximately 0.4 in European Americans but ≤0.10 in African Americans (see Stein et al. [11]). As a result, it has been difficult to determine whether the GWAS signal with SNPs in the distal *GSDMA* region are independent signals for asthma risk in European-ancestry populations, or merely a result of their LD with the core region SNPs. In our studies of children in the CREW cohorts [17], considering the alleles associated with asthma in Europeans as the effect alleles, SNPs in *GSDMA* had estimated ORs >1 in the European American sample but ORs <1 in the African American sample (Fig. 1 in [17]). We attributed this finding to the very different LD patterns between these populations but did not have sufficient power to determine whether the *GSDMA* SNPs were significantly associated with asthma in the African American sample. However, those results are consistent with the data we report here. That is, the common *GSDMA* alleles associated with reduced risk of asthma in the CREW African American cohort were associated with reduced *GSDMA* expression in lung tissue [14], in nearly all tissues in the Genotype-Tissue Expression consortium [62], and in upper airway epithelial cells in our study (Additional file 1: Table S5). These data combined with the results we report here provide a link between *GSDMA* expression and asthma risk and convergent evidence for increased expression of *GSDMA* promoting asthma pathobiology.

Notably, the high-risk haplotype 4 includes variants associated with increased expression of both *GSDMB* (i.e., rs2305480 and rs113571956) and *GSDMA* (i.e., rs11382230 and rs113571956). The increased expression of both gasdermin genes in individuals carrying haplotype 4 may account for the modifying effects of this haplotype on asthma risk and severity. For example, in a combined sample of African American children from two cohorts (APIC and URECA), the rs113282230-T allele was associated with reduced lung function, a marker of asthma severity, lower blood neutrophil count, potentially reflecting rapid turnover due to increased trafficking to mucosal surfaces in response to epithelial signals, and low total serum IgE, reflecting a T2-low asthma phenotype [63]. In an independent sample of adults with asthma, haplotype 4 was also associated with asthma severity.

While our study provides evidence for African-specific variants that modify the genetic risk attributed to the 17q12-q21 childhood-onset asthma locus by increasing expression of *GSDMA*, there are limitations. First, the novel SNPs occur exclusively on haplotypes with variants associated with increased *GSDMB* expression. Therefore, we could not determine whether the novel alleles by themselves contribute to asthma risk or whether increased expression of *GSDMA* modifies risk only in the presence of increased *GSDMB* expression. Future studies using gene editing approaches, creating isogenic cell lines with combinations of genotypes in which the shared and African-specific asthma risk variants can be decoupled, could help to differentiate these possibilities. Second, airway epithelial cell expression of *GSDMA* was too low to determine whether the combined expression levels of both genes were associated with greater risk or severity of asthma compared to expression levels of each gene separately. Nonetheless, we demonstrated that haplotype 4 was associated with asthma in Black children in the CREW cohort and asthma severity in Black adults. Using phenotype and whole-genome sequence data in two African American cohorts, we further showed that the novel variants were associated with markers of T2-low, severe asthma in children. Finally, we focused our eQTL and chromatin studies in airway epithelial cells. The rationale for this choice was that these are sentinel cells in the airway that respond to inhaled microbes, pollution, and allergens and mediate their downstream effects on asthma risk. In our earlier study [17], we showed that expression of *GSDMB* in airway epithelial cells, but not in peripheral blood cells, modulated the risk for childhood-onset asthma at the 17q12-q21 locus. However, we cannot rule out the possibility that the novel variants regulate the expression of other genes in immune cells under specific conditions that modify the features of asthma severity observed in our study.

## Conclusions

This study provides a strategy for identifying population-specific disease-associated variants at GWAS loci that were missed in previous studies. We suggest that other loci with effect sizes that differ among global populations would be amenable to such an approach and potentially yield a wealth of functional variants that are missed by current GWAS and fine-mapping approaches. The abundance of whole-genome sequence data now available in worldwide populations will facilitate the discovery of such variants and provide a rich source of novel therapeutic targets and a substrate for truly personalized medicine.

## Supplementary Information

**Additional file 1. **Contains Supplementary Methods, Supplementary Tables (Table S1-10), and Supplementary Figures (Fig. S1-14), and corresponding references. **Supplementary Methods**. Descriptions of Populations. Building Consensus Sequences in the Critical Region. **Table S1**. Characteristics of the APIC and URECA Cohorts. **Table S2**. Predicted Haplotypes in CREW. **Table S3**. Haplotype Frequencies in Whole Genome Sequences. **Table S4**. Worldwide Frequencies of African-specific SNPs. **Table S5**. cis-eQTL Results for SNPs in or near *GSDMA*. **Table S6**. ENCODE Cell Lines and DNAse Clustering at pcHi-C Region. **Table S7**. pcHi-C Target Genes for African-specific Variants in Airway Epithelial Cells. **Table S8**. pcHi-C Target Genes for African-specific Variants in Airway Immune Cells. **Table S9**. Quantitative Trait Association Results in the APIC and URECA Cohorts. **Table S10**. African American Adult Asthmatics by Severity and Genotype. **Figure S1**. Overview of Study Design. **Figure S2**. ChromoPainter Analysis. **Figure S3**. ChromoPainter Visualization of Haplotype Breakpoints. **Figure S4**. ChromoPainter Display of the 17q12-q21 Region in Haplotype 4 Homozygotes. **Figure S5**. Ancestry PCA plots for APIC and URECA Children. **Figure S6**. eQTL Box Plots of rs28623237 Genotype and *GSDMA* Expression in CAAPA2. **Figure S7**. LD Plot of African-specific Variants and SNPs in or near *GSDMA*. **Figure S8**. eQTL Box Plots of rs113282230 Genotype and *GSDMA* Expression Conditioned on *GSDMA* SNPs. **Figure S9**. eQTL Violin Plots of rs235480 and rs1132828830 Genotypes on *GSDMA* and *GSDMB* Expression. **Figure S10**. LD Plot of the African-specific Variants and SNPs in the Core Region of The 17q12-q21 Locus. **Figure S11**. Chromatin Annotations in the Region Encoding the African-specific SNPs in ENCODE Cell Lines. **Figure S12**. eGenes for rs113282230 in Immune Cells. **Figure S13**. pcHi-C Data for rs113282230 in Immune Cells. **Figure S14**. Rs113282230 Genotype Effect on Asthma Prevalence by rs2305480 AA And GG Genotypes in APIC and URECA.

**Additional file 2.** Region-wide eQTL results in airway epithelial cells from 189 African American children in the URECA cohort.

## Data Availability

The whole-genome sequences used in this study are available through dbGaP (see Table 1). The RNA-seq data for URECA children is in Gene Expression Omnibus (GEO) with accession number GSE145505 (https://www.ncbi.nlm.nih.gov/geo/query/acc.cgi?acc=GSE145505) [39]. The promoter capture Hi-C data is in GEO with accession number GSE152549 (https://www.ncbi.nlm.nih.gov/geo/query/acc.cgi?acc=GSE152549) [20].

## Abbreviations

APIC: Asthma Phenotypes in the Inner City
CAAPA: Consortium on Asthma in African-ancestry Populations in the Americas
CHiCAGO: Capture HiC Analysis of Genomic Organization
CI: Confidence interval
CREW: Children’s Respiratory and Environmental Workgroup
ECHO: Environmental Influences on Child Health Outcomes
ENCODE: Encyclopedia of DNA Elements
eQTL: Expression quantitative trait loci
FeNO: Fractional exhaled nitric oxide
FEV1: %predicted forced expiratory capacity at 1 second
FDR: False discovery rate
FVC: FEV1/forced vital capacity
*GSDMA*: Gasdermin A
*GSDMB*: Gasdermin B
GWAS: Genome-wide association studies
ICAC: Inner City Asthma Consortium
IgE: Immunoglobulin E
kb: Kilobase
LD: Linkage disequilibrium
NHB: Non-Hispanic Black
NHEK: Human epidermal keratinocytes
NHW: Non-Hispanic White
*ORMDL3*: ORM1-like 3
PC: Principal component
pcHi-C: Promoter capture Hi-C
OR: Odds ratio
SNP: Single-nucleotide polymorphism
T2: Type 2
TSS: Transcription start site
URECA: Urban Environment and Childhood Asthma
