# Supplementary material for: African-specific alleles modify risk for asthma at the 17q12-q21 locus in African Americans
Source: Genome Med. 2022 Sep 29;14:112. doi: 10.1186/s13073-022-01114-x (PMC9520885; doi:10.1186/s13073-022-01114-x)
Supplement: Supplementary file 1 — Additional file 1. Contains Supplementary Methods, Supplementary Tables (Table S1-10), and Supplementary Figures (Fig. S1-14), and corresponding references. Supplementary Methods. Descriptions of Populations. Building Consensus Sequences in the Critical Region. Table S1. Characteristics of the APIC and URECA Cohorts. Table S2. Predicted Haplotypes in CREW. Table S3. Haplotype Frequencies in Whole Genome Sequences. Table S4. Worldwide Frequencies of African-specific SNPs. Table S5. cis-eQTL Results for SNPs in or near GSDMA. Table S6. ENCODE Cell Lines and DNAse Clustering at pcHi-C Region. Table S7. pcHi-C Target Genes for African-specific Variants in Airway Epithelial Cells. Table S8. pcHi-C Target Genes for African-specific Variants in Airway Immune Cells. Table S9. Quantitative Trait Association Results in the APIC and URECA Cohorts. Table S10. African American Adult Asthmatics by Severity and Genotype. Figure S1. Overview of Study Design. Figure S2. ChromoPainter Analysis. Figure S3. ChromoPainter Visualization of Haplotype Breakpoints. Figure S4. ChromoPainter Display of the 17q12-q21 Region in Haplotype 4 Homozygotes. Figure S5. Ancestry PCA plots for APIC and URECA Children. Figure S6. eQTL Box Plots of rs28623237 Genotype and GSDMA Expression in CAAPA2. Figure S7. LD Plot of African-specific Variants and SNPs in or near GSDMA. Figure S8. eQTL Box Plots of rs113282230 Genotype and GSDMA Expression Conditioned on GSDMA SNPs. Figure S9. eQTL Violin Plots of rs235480 and rs1132828830 Genotypes on GSDMA and GSDMB Expression. Figure S10. LD Plot of the African-specific Variants and SNPs in the Core Region of The 17q12-q21 Locus. Figure S11. Chromatin Annotations in the Region Encoding the African-specific SNPs in ENCODE Cell Lines. Figure S12. eGenes for rs113282230 in Immune Cells. Figure S13. pcHi-C Data for rs113282230 in Immune Cells. Figure S14. Rs113282230 Genotype Effect on Asthma Prevalence by rs2305480 AA And GG Genotypes in APIC and URECA. [file 13073_2022_1114_MOESM1_ESM.docx]

**African-specific alleles modify risk for asthma**

**at the 17q12-q21 locus in African Americans**

Charles Washington III^1^, Matthew Dapas^1^, Arjun Biddanda^1^, Kevin M. Magnaye^1^, Ivy Aneas^1^, Britney A. Helling^1^, Brooke Szczesny^2^, Meher Preethi Boorgula^3^, Margaret A. Taub^4^, Eimear Kenny^5^, Rasika A. Mathias^2^ and Kathleen C. Barnes^3^, CAAPA^6^, Gurjit K. Khurana Hershey^7^, Carolyn M. Kercsmar^7^, Jessica D. Gereige^8^, Melanie Makhija^9^, Rebecca S. Gruchalla^10^, Michelle A. Gill^10^, Andrew H. Liu^11^, Deepa Rastogi^12^, William Busse^13^, Peter J. Gergen^14,^, Cynthia M. Visness^15^, Diane R. Gold^16^, Tina Hartert^17^, Christine C. Johnson^18^, Robert F. Lemanske, Jr.^19^, Fernando D. Martinez^20^, Rachel L. Miller^21^, Dennis Ownby^18^, Christine M. Seroogy^19^, Anne L. Wright^20^, Edward M. Zoratti^22^, Leonard B. Bacharier^23^, Meyer Kattan^24^, George T. O’Connor^25^, Robert A. Wood^26^, Marcelo A. Nobrega^1^, Matthew C. Altman^27^, Daniel J. Jackson^19^, James E. Gern^19^, Christopher G. McKennan^28^, Carole Ober^1^

^1^ Department of Human Genetics, The University of Chicago, Chicago, IL

^2^ Department of Medicine, Johns Hopkins University, Baltimore, MD

^3^ Department of Medicine, University of Colorado Denver, Aurora, CO

^4^ Department of Biostatistics, Bloomberg School of Public Health, Johns Hopkins University, Baltimore, MD

^5^ Department of Medicine, Icahn School of Medicine at Mount Sinai, New York, NY

^6^ Consortium on Asthma in African-ancestry Populations in the Americas

^7^ Division of Asthma Research, Cincinnati Children’s Hospital, Cincinnati, OH

^8^ Department of Medicine, Division of Pulmonary, Allergy, Sleep, and Critical Care Medicine, Boston University School of Medicine, Boston, MA

^9^ Ann and Robert H. Lurie Children’s Hospital of Chicago, Chicago, IL

^10^ University of Texas Southwestern Medical Center, Dallas, TX

^11^ Children's Hospital Colorado and University of Colorado School of Medicine, Aurora, CO

^12^ Children’s National Hospital and George Washington University School of Medicine and Health Sciences, Washington, DC

^13^ University of Wisconsin School of Medicine and Public Health, Madison, WI

^14^ NIH/NIAID, Bethesda MD

^15^ Rho Federal Systems Division, Inc, Durham, NC

^16^ The Channing Division of Network Medicine, Department of Medicine, Brigham and Women’s Hospital and Department of Environmental Health, Harvard T.H. Chan School of Public Health, Harvard University, Boston, MA

^17^ Department of Medicine, Vanderbilt University School of Medicine, Nashville, TN

^18^ Department of Public Health Sciences, Henry Ford Health Systems, Detroit, MI

^19^ Department of Pediatrics, University of Wisconsin School of Medicine and Public Health, Madison, WI

^20^ Asthma and Airway Disease Research Center, University of Arizona, Tucson, AZ

^21^ Department of Medicine, Division of Clinical Immunology Icahn School of Medicine at Mount Sinai, New York, NY

^22^ Department of Medicine, Henry Ford Health Systems, Detroit, MI

^23^ Department of Pediatrics, Monroe Carell Jr Children’s Hospital at Vanderbilt University Medical Center, Nashville, TN

^23^ Department of Pediatrics, Columbia University Medical Center, New York, NY

^24^ Pulmonary Center, Boston University School of Medicine, Boston, MA

^25^ Department of Pediatrics, Johns Hopkins University, Baltimore, MD

^26^ Immunology Division, Benaroya Research Institute Systems, Seattle, WA; Department of Medicine, University of Washington, Seattle, WA

^27^ Department of Statistics, University of Pittsburgh, Pittsburgh, PA

**Table of Contents**

**Supplementary Methods…………..**………………………………….……………...….……. 5

Descriptions of Populations ………………………………………………………..…... 6

Building Consensus Sequences in the Critical Region………………………………… 8

**Supplementary Tables** ……………………………………………..………………….…….. 9

Table S1. Characteristics of the APIC and URECA Cohorts .………………….…….. 10

Table S2. Predicted Haplotypes in CREW.……………………….……………..….... 11

Table S3. Haplotype Frequencies in Whole Genome Sequences ………………..…... 12

Table S4. Worldwide Frequencies of African-specific SNPs .……….…………..…... 13

Table S5. cis-eQTL Results for SNPs in or near *GSDMA*………………………..…... 14

Table S6. ENCODE Cell Lines and DNAse Clustering at pcHi-C region .……....…... 15

Table S7. pcHi-C Target Genes for African-specific Variants in Airway Epithelial Cells……………………………………………………………...……….….. 17

Table S8. pcHi-C Target Genes for African-specific Variants in Airway Immune Cells…………………………………………………………………...….….. 18

Table S9. Quantitative Trait Association Results in the APIC and URECA Cohorts ... 19

Table S10. African American Adults Asthmatics by Severity and Genotype …….….. 21

**Supplementary Figures** ……………………………………………………………………… 22

Fig S1. Overview of Study Design…………………..………………….………..….. 23

Fig S2. Chromopainter Analysis .……………………………………..………..…... 24

Fig S3. ChromoPainter Visualization of Haplotype Breakpoints .………….…..…….. 25

Fig S4. Chromopainter Display of the 17q12-q21 Region in Haplotype 4 Homozygotes……………………………………………..……….…….….. 26

Fig S5. Ancestry PCA plots for APIC and URECA Children………………….……... 27

Fig S6. eQTL Box Plots of rs28623237 Genotype and *GSDMA* Expression in CAAPA2.……………………….…………………………………..…….… 28

Fig S7. LD Plot of African-specific Variants and SNPs in or near *GSDMA*…..…….... 29

Fig S8. eQTL Box Plots of rs113282230 Genotype and *GSDMA* Expression

Conditioned on *GSDMA* SNPs .…………………………………………….. 30

Fig S9. eQTL Violin Plots of rs2305480 and rs113282230 genotypes on *GSDMA*

and *GSDMB* Expression.....…………………………………………...….…. 31

Fig S10. LD Plot of the African-specific Variants and SNPs in the Core Region

of the 17q12-q21 Locus .……………………………………………….…... 32

Fig S11. Chromatin Annotations in the Region Encoding the African-specific

SNPs in ENCODE Cell Lines…………………………..…………….....…. 33

Fig S12. eGenes for rs113282230 in Immune Cells…………….…….….………..….. 34

Fig S13. pcHi-C Data for rs113282230 in Immune Cells.………………..………..….. 35

Fig S14. rs113282230 Genotype Effect on Asthma Prevalence by rs2305480 AA

and GG Genotypes in APIC and URECA.……………………………...….. 36

**References** .…………………………………………………………..…................................... 37

**Supplementary Methods**

**Descriptions of Cohorts Used in These Studies**

1. Children’s Respiratory and Environmental Workgroup (CREW): CREW is a component study of the National Institutes of Health-funded Environmental Influences on Child Health Outcomes (ECHO) Program. CREW includes 12 U.S. longitudinal birth cohorts with asthma-related phenotypes that are diverse with respect to race, ethnicity, geographical distribution, ascertainment, and year of recruitment, which varies between 1987 and 2019 (see Table 1 in Gern et al. 2019^1^ for descriptions of the individual cohorts). Our study focused on the 1,647 Non-Hispanic White and 868 Non-Hispanic Black subjects who were at least age 6 years at their most recent visit, at which time diagnoses of asthma could be made, and were genotyped for five 17q12-q21 tag SNPs in an earlier study^2^. Asthma was based on a diagnosis based on a doctor or health-care provider diagnosis by age 6; controls were never diagnosed asthma by age 6 or at the last age at which the participant was studied. Institutional review board approval was received from all participating sites, and written informed consent was obtained from legal guardians of all participating children, who also assented to participating. The studies described in this manuscript were approved by the University of Chicago Institutional Review Board (protocol 17-1066).

The CREW participants were included in the association studies between the 17q12-q21 5-SNP haplotypes and asthma.

1. EVE Consortium: Whole genome sequences from 100 European American and 90 African American asthmatic subjects (children and adults) in the EVE Asthma Genetics Consortium, as described in Igartua et al.^3^, were included in these studies (dbGaP Study Accession phs001156.v1.p1; <https://www.ncbi.nlm.nih.gov/projects/gap/cgi-bin/study.cgi?study_id=phs001156.v2.p1>). EVE was a collaboration between U.S. investigators who had conducted genome-wide association studies (GWAS) of asthma. The main objective of EVE was to combine results of individual studies to increase the overall power to identify loci for asthma and asthma-associated traits^3-7^. The consortium included investigators at 9 U.S. institutions with GWAS results for >10,000 individuals representing European American, African American, African Caribbean, U.S. Hispanic, and Mexican populations. All cases had a doctor’s diagnosis of asthma. The studies in this manuscript were approved by the University of Chicago Institutional Review Board (protocol 6626).

The EVE participants were included in the sequencing studies to further refine 17q12-q21 haplotype lengths, define a critical region, and identify novel variants on the high-risk haplotype.

1. Consortium on Asthma in African-ancestry Populations in the Americas (CAAPA): Whole genome sequences from 168 asthmatic and 152 non-asthmatic African American subjects (both children and adults) in CAAPA were included in our study (dbGaP Study Accession phs001123.v1.p1; <https://www.ncbi.nlm.nih.gov/projects/gap/cgi-bin/study.cgi?study_id=phs001123.v2.p1>). Asthmatic individuals of African descent tend to have more severe asthma and more clinical symptoms than individuals of European ancestries. At the time that CAAPA was initiated, coverage of common genetic markers in public databases and on commercially available SNP chips has been inadequate to detect and measure genetic associations among African admixed populations. The aim of CAAPA was to catalog genetic diversity in populations of African descent, especially those whose ancestry reflects the African Diaspora in the Americas. All cases had a doctor’s diagnosis of asthma. The utilization of CAAPA sequences in this manuscript were approved by the University of Chicago Institutional Review Board (protocol 19-0377-CR001).

The CAAPA participants were included in the sequencing studies to refine 17q12-q21 haplotype lengths, define a critical region, and identify novel variants on the high-risk haplotype. Individuals with gene expression data in nasal epithelial cells were included in the eQTL validation/replication studies of *GSDMA*.

1. Asthma Phenotypes in the Inner City (APIC): Whole genome sequences were generated by our group^8^ from asthmatic children in the APIC study^9^; this study included the 321 children who were parent-identified as non-Hispanic Black. All sequences have been deposited in dbGaP (dbGaP Study Accession phs002921.v1.p1; [http://www.ncbi.nlm.nih.gov/projects/gap/cgi-bin/study.cgi?study_id=phs002921.v1.p1](https://urldefense.com/v3/__http:/www.ncbi.nlm.nih.gov/projects/gap/cgi-bin/study.cgi?study_id=phs002921.v1.p1__;!!MvNZe7V6M35iZPhbgng-hfU!0V6Od-mXpSafX7pJMBXK-9kifnzn2KPHnd9J1zgkeomagpVCronb-EBtKXZE-C8j_vGiUdpUaGgx8Bj7bjH2cOgBTgan53kF$)). APIC, a component study of the Inner City Asthma Consortium (ICAC)^10^. was a 1-year, prospective, epidemiological investigation of children and adolescents with asthma (ages 6-17) living in low-income areas (≥20% of residents below poverty level) in nine U.S. cities (Baltimore, MD; Boston, MA; Chicago, IL; Cincinnati, OH; Dallas, TX; Denver, CO; Detroit, MI; New York, NY; Washington, DC). The APIC participants were required to have a diagnosis of asthma by a physician and to have had at least two episodes requiring bronchodilator administration within the previous year. African American ancestries in the children included in these studies were determined by ancestry PCs (**Additional File 1: Fig. S5**). Institutional review board approval was received from all participating sites, and written informed consent was obtained from legal guardians of all participating children, who also assented to participating. The studies described in this manuscript were approved by the University of Chicago Institutional Review Board (protocol 19-0046). The clinical studies were approved by IRBs at each of the nine participating centers.

The APIC participants were included in the sequencing studies to refine 17q12-q21 haplotype lengths, define a critical region, and identify novel variants on the high-risk haplotype. This sample was also included in the association studies between the African-specific variants and clinical phenotypes. See **Additional File 1: Table S1** for a description of the clinical characteristics of this cohort.

1. Urban Environment and Childhood Asthma (URECA): Whole genome sequences were generated by our group^8^ from children in the URECA study^11^; this study included the 176 asthmatic and 153 non-asthmatic children who were parent-identified as non-Hispanic Black. All sequences have been deposited in dbGaP (dbGaP Study Accession phs002921.v1.p1; ; [http://www.ncbi.nlm.nih.gov/projects/gap/cgi-bin/study.cgi?study_id=phs002921.v1.p1](https://urldefense.com/v3/__http:/www.ncbi.nlm.nih.gov/projects/gap/cgi-bin/study.cgi?study_id=phs002921.v1.p1__;!!MvNZe7V6M35iZPhbgng-hfU!0V6Od-mXpSafX7pJMBXK-9kifnzn2KPHnd9J1zgkeomagpVCronb-EBtKXZE-C8j_vGiUdpUaGgx8Bj7bjH2cOgBTgan53kF$)). URECA, a component study of ICAC^10^, enrolled pregnant women living in low-income areas of four U.S. cities (Baltimore, MD; Boston, MA; New York, NY; St. Louis, MO) who reported that one or both parents of the index pregnancy had a history of asthma or allergic diseases. This prospective, longitudinal study followed each child through adolescence, periodically collecting samples and clinical and environmental exposure data. At the age 11 visit, nasal swabs were collected from children for gene expression studies, as described^12^ (RNA-seq data deposited in GEO GSE145505). Diagnoses of asthma were based on doctor’s diagnosis, lung function reversibility, or symptom recurrence^13^. African American ancestries in the children included in these studies were determined by ancestry PCs (**Additional File 1: Fig. S5**). Institutional review board approval was received from all participating sites, and written informed consent was obtained from legal guardians of all participating children, who also assented to participating in the studies. The studies described in this manuscript were approved by the University of Chicago Institutional Review Board (protocol 19-0046). The clinical studies were approved under a Central IRB at the University of Wisconsin and WIRB IRB # 20142570.

The URECA participants were included in the sequencing studies to refine 17q12-q21 haplotype lengths, define a critical region, and identify novel variants on the high-risk haplotype. A subset of this sample (n=189) was also included in the eQTL studies and the association studies between the African-specific variants and clinical phenotypes. See **Additional File 1: Table S1** for a description of the clinical characteristics of this cohort.

1. Chicago Asthma Genetic Studies (CAG). Adults (≥18 years) with and without asthma were recruited for genetic studies from asthma clinics and through recruitment posters throughout the University of Chicago Medical Center between 2010 and 2014^14,15^. All subjects were clinically evaluated, and asthma status was confirmed by pulmonary function and methacholine challenge or reversibility studies. Asthma severity scores were assigned based on medication (steroid) use and lung function measures^16^. Subjects were considered to be African American if at least three of their four grandparents were considered by the subject to be African American or African. Blood DNA was genotyped with Illumina arrays and imputed using the 1,000 genomes phase 3 reference panel, as described previously^15^. Genotypes for 5 SNPs at the 17q12-q21 locus were extracted for these studies. This study included 63 African American CAG subjects with a diagnosis of asthma. These studies were approved by the University of Chicago Institutional Review Board (protocols 09-421-B and 153651A).

This sample was included in the studies of asthma severity and the 17q12-21 5-SNP high-risk haplotype.

**Building Consensus Sequences of the Critical Region**

We created consensus sequences of the critical region in individuals who were homozygous for one of the six haplotypes: the shared protective haplotype in European Americans (2n=22 chromosomes) and in African Americans (2n=14 chromosomes) – haplotype 1 in manuscript **Fig. 1**, the shared risk haplotype in European Americans (2n=66 chromosomes) and African Americans (2n=260 chromosomes) – haplotype 2 in manuscript **Fig. 1**, the African American-specific high-risk haplotype (2n=36 chromosomes) – haplotype 4 in manuscript **Fig. 1**, and an additional African-American specific haplotypes with uncertain risk status (2n=40) – haplotype 3 in manuscript **Fig. 1**. The consensus sequences were built using the most frequent nucleotide at each position. When the most frequent nucleotide had a frequency of 0.5 or less, standard codes from the International Union of Pure and Applied Chemistry were used^17^. Next, pairwise sequence alignments were conducted using the EMBOSS Needle Alignment Tool^18^. For each alignment, the following parameters were used to prevent formation of gaps because all sequences were of the same defined region and therefore perfectly aligned: Gap Open Penalty 100, Gap Extend Penalty 10, End Gap Penalty: False, End Gap Open 100, and End Gap Extend Penalty 10.

**Supplementary Tables**

**Table S1.** Characteristics of the APIC and URECA cohorts.

| **Characteristic** | **Combined** | **APIC** | **URECA** |
| --- | --- | --- | --- |
| Sample size | 613 | 319 | 294 |
| Age, years | 10 (9-11) | 11 (9-14) | 10 (9-10) |
| Female sex | 46% | 42% | 50% |
| *Ancestry (mean admixture)* |  |  |  |
| African | 81.4% | 81.6% | 81.2% |
| European | 15.9% | 15.8% | 16.1% |
| East Asian/Native American | 2.7% | 2.6% | 2.7% |
| *Clinical Phenotypes* |  |  |  |
| Asthma | 444 (72%) | 319 (100%) | 125 (43%) |
| Eczema diagnosis | 243 (40%) | 188 (59%) | 55 (19%) |
| FEV_1_, % predicted | 96.5 (87.1-107.4) | 92.4 (83.3-105.2) | 100.3 (92.2-108.4) |
| Bronchodilator response | 8.9 (3.9-14.8) | 11.3 (5.5-18.7) | 6.5 (2.7-11.2) |
| FEV/FVC | 0.81 (0.75-0.86) | 0.78 (0.70-0.84) | 0.84 (0.79-0.88) |
| FeNO, ppb | 18 (10-36) | 20.5 (13-40) | 10 (6-24) |
| Total serum IgE, kU/L | 170.5 (51-504.5) | 283.5 (90.5-840.5) | 109 (36-273) |
| Blood eosinophil count, cells/μL | 210 (100-400) | 300 (150-481) | 200 (100-315) |
| Blood neutrophil count, cells/μL | 2500 (1800-3490) | 2600 (1800-3600) | 2400 (1800-3300) |

**Table S2.** Predicted haplotypes in CREW European American and African American subjects. Predicted haplotypes with estimated frequencies less than ½_N_ for each sample size are reported as 0. Haplotypes are ordered by the numbers assigned by haplo.em^19^ (see Methods).

| **Haplotype** | **Haplotype Motif** | **European American** | | **African American** | |
| --- | --- | --- | --- | --- | --- |
|  |  | **Frequency in Controls (2n=2,694)** | **Frequency in Cases (2n=600)** | **Frequency in Controls (2n=1,100)** | **Frequency in Cases (2n=636)** |
| 1 | GACAG | 0.43 | 0.37 | 0.14 | 0.10 |
| 2 | CGTGA | 0.48 | 0.55 | 0.45 | 0.41 |
| 3 | GGTGA | 0.0072 | 0.012 | 0.16 | 0.17 |
| 4 | GGTAA | 0.0040 | 0.0088 | 0.13 | 0.18 |
| 5 | CGTAA | 0.0012 | -- | 0.028 | 0.044 |
| 6 | GGCAA | 0.040 | 0.036 | 0.026 | 0.032 |
| 7 | GGCAG | 0.00069 | -- | 0.029 | 0.028 |
| 8 | GACGG | 0.016 | 0.012 | 0.023 | 0.016 |
| 9 | CGCAG | 0.0077 | 0.0050 | 0.0041 | 0.0066 |
| 10 | CGCAA | -- | -- | -- | -- |
| 11 | CGTGG | 0.00055 | -- | -- | 0 |
| 12 | GATGA | 0.00037 | -- | -- | 0.0033 |
| 13 | CGTAG | 0.00078 | 0.0017 | 0.0020 | 0.0049 |
| 14 | GGCGA | 0 | -- | -- | 0 |
| 15 | CGCGG | -- | -- | 0 | -- |
| 16 | GACAA | 0.00075 | -- | -- | -- |
| 17 | GACGA | 0.0026 | 0.0034 | 0.00091 | -- |
| 18 | GATAA | -- | -- | 0.00380 | 0 |
| 19 | GGCGG | -- | -- | 0 | -- |
| 20 | GGTAG | 0.00038 | 0 | 0.0018 | 0 |
| 21 | CACAG | -- | -- | 0.0013 | -- |
| 22 | CATGA | -- | -- | -- | -- |
| 23 | CACGA | -- | -- | 0 | -- |
| 24 | CACGG | -- | -- | 0 | -- |

**Table S3.** Haplotype frequencies in the whole genome sequence dataset.

| **Haplotype** | **EVE EA (2n=200)** | **EVE AA (2n=180)** | **APIC AA (2n=642)** | **CAAPA AA (2n=640)** | **URECA AA (2n=658)** |
| --- | --- | --- | --- | --- | --- |
| Haplotype 1: GACAG | 0.35 | 0.13 | 0.086 | 0.11 | 0.091 |
| Haplotype 2: CGTGA | 0.59 | 0.33 | 0.42 | 0.44 | 0.43 |
| Haplotype 3: GGTGA | 0.01 | 0.21 | 0.20 | 0.18 | 0.19 |
| Haplotype 4: GGTAA | - | 0.16 | 0.15 | 0.14 | 0.16 |
| Haplotype 5: CGTAA | - | 0.050 | 0.030 | 0.019 | 0.037 |
| Haplotype 6: GGCAA | 0.025 | 0.050 | 0.023 | 0.044 | 0.032 |
| Haplotype 7: GGCAG | - | 0.044 | 0.042 | 0.023 | 0.023 |
| Haplotype 8: GACGG | 0.020 | 0.017 | 0.014 | 0.020 | 0.020 |
| Haplotype 9: CGCAG | 0.0050 | - | 0.011 | 0.0063 | 0.0076 |
| Haplotype 10: CGCAA | - | 0.0056 | - | 0.0031 | 0.0015 |
| Haplotype 11: CGTGG | - | 0.0056 | - | 0.0016 | - |
| Haplotype 12: GATGA | - | - | 0.0031 | 0.0031 | 0.0015 |
| Haplotype 13: CGTAG | - | - | 0.0031 | - | 0.0061 |
| Haplotype 14: GGCGA | 0.0050 | - | - | 0.0016 | - |
| Haplotype 15: CGCGG | - | - | - | - | 0.0015 |
| Haplotype 16: GACAA | - | - | 0.0031 | - | 0.0015 |
| Haplotype 17: GACGA | - | - | 0.0016 | - | - |
| Haplotype 18: GATAA | - | - | 0.0047 | - | 0.0015 |
| Haplotype 19: GGCGG | - | - | 0.0016 | - | - |
| Haplotype 20: GGTAG | - | - | 0.0031 | - | 0.0015 |
| Haplotype 21: CACAG | - | - | - | - | - |

**Table S4.** Frequencies of the nine African-specific SNPs in the Haplotype 4 critical region in worldwide populations.

| **Variant Description** | | | **Worldwide Populations (dbSNP)** | | | | | | | | | **UK Biobank** | |
| --- | --- | --- | --- | --- | --- | --- | --- | --- | --- | --- | --- | --- | --- |
| **rsID** | **Variant** | **Position (hg38)** | **European** | **African** | **African American** | **Asian** | **East Asian** | **Other Asian** | **Latin American 1** | **Latin American 2** | **South Asian** | **White** | **Non-White** |
| rs150276395 | G->A | chr17:39908449 | 0.0010 | 0.13 | 0.13 | 0 | 0 | 0 | 0.034 | 0.02 | 0 | 0.00068 | 0.018 |
| rs8065520 | T->C | chr17:39915395 | 0.0053 | 0.24 | 0.24 | 0 | 0 | 0 | 0.070 | 0.026 | 0 | 0.0015 | 0.040 |
| rs73985226 | A->G | chr17:39920081 | 0.00027 | 0.17 | 0.18 | 0 | 0 | 0 | 0 | 0.002 | 0 | 0.00053 | 0.017 |
| rs73985227 | G->A | chr17:39921437 | 0.0011 | 0.12 | 0.12 | 0 | 0 | 0 | 0.017 | 0.017 | 0 | 0.00053 | 0.018 |
| rs28623237 | T->C | chr17:39924694 | 0.0011 | 0.16 | 0.16 | 0 | 0 | 0 | 0.027 | 0.021 | 0 | 0.00059 | 0.024 |
| rs73985229 | G->A | chr17:39925400 | 0.0016 | 0.12 | 0.12 | 0 | 0 | 0 | 0.018 | 0.011 | 0 | 0.00055 | 0.018 |
| rs113282230 | A->T | chr17:39927157 | 0.0014 | 0.12 | 0.12 | 0 | 0 | 0 | 0.021 | 0.016 | 0 | 0.00055 | 0.018 |
| rs113571956 | A->T | chr17:39927234 | 0.0014 | 0.12 | 0.12 | 0 | 0 | 0 | 0.021 | 0.016 | 0 | 0.00055 | 0.018 |
| rs73985230 | T->C | chr17:39929476 | 0.0015 | 0.11 | 0.12 | 0 | 0 | 0 | 0.019 | 0.012 | 0 | 0.00054 | 0.016 |

**Table S5.** cis-eQTL mapping results for 25 SNPs in or near *GSDMA* that are shown in **Additional File 1: Fig. S7**. Gene expression data are from upper airway epithelial cells from 189 African American children from the URECA cohort.

| **SNP-Effect Allele** | **Position (hg38)** | **Gene** | **Statistic** | **P-Value** | **FDR** | **Beta** |
| --- | --- | --- | --- | --- | --- | --- |
| rs7222154-T | chr17:39933720 | GSDMA | -3.40 | 0.00084 | 0.230 | -0.65 |
| rs56199421-T | chr17:39934555 | GSDMA | -3.81 | 0.00020 | 0.095 | -0.70 |
| rs8079416-C | chr17:39936460 | GSDMA | -3.46 | 0.00068 | 0.200 | -0.66 |
| rs35123741-G | chr17:39936677 | GSDMA | -3.42 | 0.00079 | 0.220 | -0.65 |
| rs12603481-A | chr17:39937086 | GSDMA | -3.46 | 0.00068 | 0.200 | -0.66 |
| rs6503525-C | chr17:39938921 | GSDMA | -3.46 | 0.00068 | 0.200 | -0.66 |
| rs7216564-A | chr17:39940919 | GSDMA | -3.46 | 0.00068 | 0.200 | -0.66 |
| rs4795406-C | chr17:39943881 | GSDMA | -3.86 | 0.00016 | 0.082 | -0.73 |
| rs4065986-A | chr17:39946388 | GSDMA | -3.68 | 0.00032 | 0.130 | -0.69 |
| rs4795408-A | chr17:39951374 | GSDMA | -3.42 | 0.00080 | 0.220 | -0.68 |
| rs8081462-C | chr17:39955937 | GSDMA | -3.78 | 0.00022 | 0.100 | -0.72 |
| rs12451084-T | chr17:39963504 | GSDMA | -3.73 | 0.00026 | 0.110 | -0.73 |
| rs3894194-A | chr17:39965740 | GSDMA | -3.51 | 0.00059 | 0.190 | -0.70 |
| rs4082190-T | chr17:39969638 | GSDMA | -3.66 | 0.00034 | 0.130 | -0.66 |
| rs59269632-G | chr17:39969978 | GSDMA | -3.43 | 0.00076 | 0.220 | -0.65 |
| rs4488488-C | chr17:39970206 | GSDMA | -3.63 | 0.00038 | 0.150 | -0.69 |
| rs4239225-A | chr17:39970859 | GSDMA | -3.40 | 0.00084 | 0.230 | -0.63 |
| rs3859192-T | chr17:39972395 | GSDMA | -3.40 | 0.00084 | 0.230 | -0.63 |
| rs3859191-A | chr17:39972461 | GSDMA | -3.40 | 0.00084 | 0.230 | -0.63 |
| rs4065876-A | chr17:39973253 | GSDMA | -3.40 | 0.00084 | 0.230 | -0.63 |
| rs60137005-T | chr17:39973743 | GSDMA | -3.40 | 0.00084 | 0.230 | -0.63 |
| rs56326707-T | chr17:39973886 | GSDMA | -3.40 | 0.00084 | 0.230 | -0.63 |
| rs56030650-A | chr17:39974934 | GSDMA | -3.40 | 0.00084 | 0.230 | -0.63 |
| rs60134943-T | chr17:39977539 | GSDMA | -3.40 | 0.00084 | 0.230 | -0.63 |
| rs3907022-C | chr17:39978636 | GSDMA | -3.40 | 0.00084 | 0.230 | -0.63 |

**Table S6.** 93 cell lines with DNAse clustering that overlaps the pcHi-C region shown in **Fig. 3B**. Rows are sorted in descending order by signal.

| **No.** | **Signal** | **CellType** | **Treatment** | **Lab** |
| --- | --- | --- | --- | --- |
| 67 | 276.5 | Jurkat | None | UW |
| 57 | 94 | HSMMtube | None | AWG |
| 91 | 84 | Th1 | None | AWG |
| 8 | 73 | Caco-2 | None | UW |
| 18 | 63.5 | GM12865 | None | UW |
| 12 | 57 | CMK | None | UW |
| 26 | 57 | H1-hESC | None | AWG |
| 38 | 56 | HepG2 | None | AWG |
| 17 | 52 | GM12864 | None | UW |
| 33 | 51 | HCPEpiC | None | UW |
| 2 | 50.67 | Adult_CD4_Th0 | None | UW |
| 70 | 47 | LNCaP | None | AWG |
| 64 | 46 | iPS | None | Duke |
| 9 | 45 | CD34+_Mobilized | None | UW |
| 31 | 44 | HCM | None | UW |
| 36 | 42.33 | HeLa-S3 | None | AWG |
| 21 | 39.5 | GM12892 | None | Duke |
| 23 | 38.5 | GM19238 | None | Duke |
| 42 | 36.67 | HMEC | None | AWG |
| 77 | 35.5 | NHDF-Ad | None | UW |
| 41 | 34.5 | HL-60 | None | UW |
| 20 | 33.67 | GM12891 | None | Duke |
| 40 | 33 | HIPEpiC | None | UW |
| 80 | 32.67 | Osteobl | None | Duke |
| 68 | 32 | K562 | None | AWG |
| 13 | 31 | Fibrobl | None | Duke |
| 73 | 30.5 | Medullo | None | Duke |
| 69 | 30 | LNCaP | androgen | Duke |
| 29 | 29 | HAc | None | UW |
| 49 | 29 | HPAF | None | UW |
| 25 | 28.67 | GM19240 | None | Duke |
| 76 | 28 | NB4 | None | UW |
| 19 | 27.33 | GM12878 | None | AWG |
| 5 | 27 | AoAF | None | UW |
| 22 | 26.5 | GM18507 | None | Duke |
| 10 | 26 | Chorion | None | Duke |
| 61 | 26 | Huh-7.5 | None | Duke |
| 89 | 26 | Stellate | None | Duke |
| 92 | 25.5 | Th2 | None | UW |
| 24 | 25 | GM19239 | None | Duke |
| 82 | 25 | pHTE | None | Duke |
| 1 | 24.33 | A549 | None | AWG |
| 37 | 24 | Hepatocytes | None | Duke |
| 62 | 24 | HUVEC | None | AWG |
| 79 | 23.67 | NHEK | None | AWG |
| 15 | 23.5 | Gliobla | None | Duke |
| 56 | 23 | HSMM | None | AWG |
| 65 | 23 | Ishikawa | 4OHTAM_100nM_30m | Duke |
| 60 | 21.5 | Huh-7 | None | Duke |
| 32 | 21 | HConF | None | UW |
| 43 | 21 | HMVEC-dBl-Ad | None | UW |
| 35 | 20 | HeLa-S3 | IFNa4h | Duke |
| 66 | 20 | Ishikawa | Estradiol_100nM_1hr | Duke |
| 34 | 19 | HEEpiC | None | UW |
| 81 | 19 | PanIsletD | None | Duke |
| 84 | 19 | ProgFib | None | Duke |
| 85 | 19 | RWPE1 | None | Duke |
| 74 | 18.5 | Melano | None | Duke |
| 90 | 18.5 | T-47D | None | Duke |
| 46 | 18 | HMVEC-LBl | None | UW |
| 75 | 18 | Myometr | None | Duke |
| 93 | 17.5 | Urothelia | None | Duke |
| 11 | 17 | CLL | None | Duke |
| 27 | 17 | H9ES | None | Duke |
| 50 | 17 | HPDE6-E6E7 | None | Duke |
| 6 | 16 | AoSMC | serum_free_media | Duke |
| 7 | 16 | BE2_C | None | UW |
| 45 | 16 | HMVEC-dNeo | None | UW |
| 47 | 16 | HMVEC-LLy | None | UW |
| 71 | 16 | MCF-7 | Hypoxia_LacAcid | Duke |
| 86 | 16 | SAEC | None | UW |
| 53 | 15 | HRCEpiC | None | UW |
| 55 | 15 | HRPEpiC | None | UW |
| 3 | 14 | AG09319 | None | UW |
| 16 | 14 | GM06990 | None | UW |
| 44 | 14 | HMVEC-dLy-Ad | None | UW |
| 58 | 14 | HSMM_emb | None | Duke |
| 14 | 13 | FibroP | None | Duke |
| 54 | 13 | HRE | None | UW |
| 59 | 13 | HTR8svn | None | Duke |
| 78 | 13 | NHDF-neo | None | UW |
| 88 | 13 | SKMC | None | UW |
| 4 | 12 | AG10803 | None | UW |
| 28 | 12 | HA-h | None | UW |
| 39 | 12 | HGF | None | UW |
| 52 | 12 | HPF | None | UW |
| 63 | 12 | HVMF | None | UW |
| 30 | 11 | HBMEC | None | UW |
| 72 | 11 | MCF-7 | None | AWG |
| 83 | 11 | PrEC | None | UW |
| 87 | 11 | SK-N-SH_RA | None | UW |
| 51 | 10 | HPdLF | None | UW |
| 48 | 8 | HNPCEpiC | None | UW |

**Table S7.** pcHi-C target genes for the nine African-specific variants and their target genes in airway epithelial cells. A search was performed using the SNP coordinate ±1,000 bp.

| **rsID** | **COORDINATES (hg19)** | **GENE OF INTERACTION** |
| --- | --- | --- |
| rs150276395 | chr17: 38064702 38064703 | *GRB7* |
| rs8065520 | chr17: 38071648 38071649 | *GRB7* |
| rs73985226 | chr17: 38076334 38076335 | *PPP1R1B; PNMT* |
| rs73985227 | chr17: 38077690 38077691 | *PPP1R1B; PGAP3; ERBB2* |
| rs28623237 | chr17: 38080947 38080948 | *GRB7; ZPBP2; PSMD3* |
| rs73985229 | chr17: 38081653 38081654 | *CDK2; GRB7; IKZF3; PSMD3; RARA* |
| rs113282230 | chr17 38083410 38083411 | *PLXDC1; CDK12; GRB7; IKZF3; PSMD3; RARA; ERBB2; PGAP3; CSF3; NR1D1; STARD3, MIEN1;* ***GSDMA****; MSL1* |
| rs113571956 | chr17: 38083487 38083488 | *PLXDC1; CDK12; GRB7; IKZF3; PSMD3; RARA; ERBB2; PGAP3; CSF3; NR1D1; STARD3, MIEN1;* ***GSDMA****; MSL1* |
| rs73985230 | chr17: 38085729 38085730 | *GRB7; STARD3; ERBB2; MIEN1;* ***GSDMA****; PSMD3; MSL1; WIPF2* |

**Table S8.** pcHi-C target genes with the nine African-specific SNPs in immune cells (from Open Target: (<https://genetics.opentargets.org/variant/17_39927157_A_T>). Aggregated scores represent the position in the quantile distribution of interaction scores across immune cell types (1 is the strongest interaction). n.i, variant not included; blank cell, no interaction. See also **Additional File 1: Fig. S13**.

|  | **pcHi-C Interactions in immune cells (aggregate scores)** | | | | | | |
| --- | --- | --- | --- | --- | --- | --- | --- |
| **rsID** | ***PPP1R1B*** | ***PGAP3*** | ***ERBB2*** | ***MIEN1*** | ***GRB7*** | ***IKZF3*** | ***ZPBP2*** |
| rs150276395 | ni | ni | ni | ni | ni | ni | ni |
| rs8065520 | ni | ni | ni | ni | ni | ni | ni |
| rs73985226 | 1 | 0.1 | 0.5 | 0.6 | 0.4 | 0.7 | 0.4 |
| rs73985227 | 1 | 0.1 | 0.5 | 0.6 | 0.4 | 0.7 | 0.4 |
| rs28623237 | 1 | 0.1 | 0.5 | 0.6 | 0.4 | 0.7 | 0.4 |
| rs73985229 | 1 | 0.1 | 0.5 | 0.6 | 0.4 | 0.7 | 0.4 |
| rs113282230 | 0.9 | 0.1 | 0.1 | 0.4 |  | 0.5 |  |
| rs113571956 | 0.9 | 0.1 | 0.1 | 0.4 |  | 0.5 |  |
| rs73985230 | 0.9 | 0.1 | 0.1 | 0.4 |  | 0.5 |  |

**Table S9.** African American quantitative trait results in the APIC/URECA sample.

| **rsID** | **Position (hg38)** | **Phenotype** | **n** | **Ref** | **Alt** | **MAF** | **Beta** | **SE** | **P_wald** |
| --- | --- | --- | --- | --- | --- | --- | --- | --- | --- |
| rs150276395 | chr17:39908449 | FEV1 (% predicted) | 606 | G | A | 0.149 | -0.1908 | 0.07902 | 1.60E-02 |
| rs8065520 | chr17:39915395 | FEV1 (% predicted) | 607 | T | C | 0.27 | -0.08965 | 0.0647 | 1.66E-01 |
| rs73985226 | chr17:39920081 | FEV1 (% predicted) | 607 | A | G | 0.124 | -0.1819 | 0.08429 | 3.13E-02 |
| rs73985227 | chr17:39921437 | FEV1 (% predicted) | 607 | G | A | 0.131 | -0.2022 | 0.08249 | 1.45E-02 |
| rs28623237 | chr17:39924694 | FEV1 (% predicted) | 607 | T | C | 0.195 | -0.1424 | 0.07064 | 4.43E-02 |
| rs73985229 | chr17:39925400 | FEV1 (% predicted) | 607 | G | A | 0.132 | -0.214 | 0.08173 | 9.06E-03 |
| rs113282230 | chr17:39927157 | FEV1 (% predicted) | 607 | A | T | 0.132 | -0.214 | 0.08173 | 9.06E-03 |
| rs113571956 | chr17:39927234 | FEV1 (% predicted) | 607 | A | T | 0.132 | -0.214 | 0.08173 | 9.06E-03 |
| rs73985230 | chr17:39929476 | FEV1 (% predicted) | 607 | T | C | 0.125 | -0.1946 | 0.08349 | 2.01E-02 |
| rs150276395 | chr17:39908449 | FEV/FVC Z-score | 600 | G | A | 0.147 | -0.07128 | 0.08473 | 4.01E-01 |
| rs8065520 | chr17:39915395 | FEV/FVC Z-score | 601 | T | C | 0.269 | -0.001346 | 0.06925 | 9.84E-01 |
| rs73985226 | chr17:39920081 | FEV/FVC Z-score | 601 | A | G | 0.125 | -0.04624 | 0.09005 | 6.08E-01 |
| rs73985227 | chr17:39921437 | FEV/FVC Z-score | 601 | G | A | 0.131 | -0.0678 | 0.08822 | 4.42E-01 |
| rs28623237 | chr17:39924694 | FEV/FVC Z-score | 601 | T | C | 0.196 | -0.08687 | 0.07538 | 2.50E-01 |
| rs73985229 | chr17:39925400 | FEV/FVC Z-score | 601 | G | A | 0.132 | -0.07255 | 0.08742 | 4.07E-01 |
| rs113282230 | chr17:39927157 | FEV/FVC Z-score | 601 | A | T | 0.132 | -0.07255 | 0.08742 | 4.07E-01 |
| rs113571956 | chr17:39927234 | FEV/FVC Z-score | 601 | A | T | 0.132 | -0.07255 | 0.08742 | 4.07E-01 |
| rs73985230 | chr17:39929476 | FEV/FVC Z-score | 601 | T | C | 0.126 | -0.05158 | 0.0892 | 5.63E-01 |
| rs150276395 | chr17:39908449 | Bronchodilator Response | 587 | G | A | 0.147 | 0.109 | 0.08444 | 1.97E-01 |
| rs8065520 | chr17:39915395 | Bronchodilator Response | 588 | T | C | 0.269 | 0.03353 | 0.06875 | 6.26E-01 |
| rs73985226 | chr17:39920081 | Bronchodilator Response | 588 | A | G | 0.124 | 0.07314 | 0.08924 | 4.13E-01 |
| rs73985227 | chr17:39921437 | Bronchodilator Response | 588 | G | A | 0.131 | 0.09179 | 0.08737 | 2.94E-01 |
| rs28623237 | chr17:39924694 | Bronchodilator Response | 588 | T | C | 0.195 | 0.06359 | 0.07461 | 3.94E-01 |
| rs73985229 | chr17:39925400 | Bronchodilator Response | 588 | G | A | 0.132 | 0.09478 | 0.08657 | 2.74E-01 |
| rs113282230 | chr17:39927157 | Bronchodilator Response | 588 | A | T | 0.132 | 0.09478 | 0.08657 | 2.74E-01 |
| rs113571956 | chr17:39927234 | Bronchodilator Response | 588 | A | T | 0.132 | 0.09478 | 0.08657 | 2.74E-01 |
| rs73985230 | chr17:39929476 | Bronchodilator Response | 588 | T | C | 0.125 | 0.0766 | 0.08838 | 3.86E-01 |
| rs150276395 | chr17:39908449 | FeNO | 423 | G | A | 0.147 | -0.07763 | 0.09859 | 4.31E-01 |
| rs8065520 | chr17:39915395 | FeNO | 423 | T | C | 0.267 | -0.000848 | 0.07975 | 9.92E-01 |
| rs73985226 | chr17:39920081 | FeNO | 423 | A | G | 0.122 | -0.08578 | 0.1052 | 4.15E-01 |
| rs73985227 | chr17:39921437 | FeNO | 423 | G | A | 0.129 | -0.08874 | 0.1026 | 3.88E-01 |
| rs28623237 | chr17:39924694 | FeNO | 423 | T | C | 0.186 | -0.04631 | 0.09041 | 6.09E-01 |
| rs73985229 | chr17:39925400 | FeNO | 423 | G | A | 0.129 | -0.06967 | 0.1016 | 4.93E-01 |
| rs113282230 | chr17:39927157 | FeNO | 423 | A | T | 0.129 | -0.06967 | 0.1016 | 4.93E-01 |
| rs113571956 | chr17:39927234 | FeNO | 423 | A | T | 0.129 | -0.06967 | 0.1016 | 4.93E-01 |
| rs73985230 | chr17:39929476 | FeNO | 423 | T | C | 0.122 | -0.06581 | 0.104 | 5.27E-01 |
| rs150276395 | chr17:39908449 | Total IgE | 603 | G | A | 0.149 | -0.1141 | 0.07844 | 1.46E-01 |
| rs8065520 | chr17:39915395 | Total IgE | 604 | T | C | 0.268 | -0.07091 | 0.06436 | 2.71E-01 |
| rs73985226 | chr17:39920081 | Total IgE | 604 | A | G | 0.125 | -0.1596 | 0.08334 | 5.59E-02 |
| rs73985227 | chr17:39921437 | Total IgE | 604 | G | A | 0.132 | -0.1764 | 0.08169 | 3.12E-02 |
| rs28623237 | chr17:39924694 | Total IgE | 604 | T | C | 0.195 | -0.08404 | 0.07008 | 2.31E-01 |
| rs73985229 | chr17:39925400 | Total IgE | 604 | G | A | 0.132 | -0.1651 | 0.08102 | 4.21E-02 |
| rs113282230 | chr17:39927157 | Total IgE | 604 | A | T | 0.132 | -0.1651 | 0.08102 | 4.21E-02 |
| rs113571956 | chr17:39927234 | Total IgE | 604 | A | T | 0.132 | -0.1651 | 0.08102 | 4.21E-02 |
| rs73985230 | chr17:39929476 | Total IgE | 604 | T | C | 0.126 | -0.1481 | 0.08261 | 7.34E-02 |
| rs150276395 | chr17:39908449 | Blood Eosinophil Count | 605 | G | A | 0.149 | -0.08234 | 0.07941 | 3.00E-01 |
| rs8065520 | chr17:39915395 | Blood Eosinophil Count | 606 | T | C | 0.269 | -0.03312 | 0.06509 | 6.11E-01 |
| rs73985226 | chr17:39920081 | Blood Eosinophil Count | 606 | A | G | 0.125 | -0.1065 | 0.08435 | 2.07E-01 |
| rs73985227 | chr17:39921437 | Blood Eosinophil Count | 606 | G | A | 0.131 | -0.1072 | 0.08271 | 1.96E-01 |
| rs28623237 | chr17:39924694 | Blood Eosinophil Count | 606 | T | C | 0.194 | -0.05196 | 0.0709 | 4.64E-01 |
| rs73985229 | chr17:39925400 | Blood Eosinophil Count | 606 | G | A | 0.132 | -0.1084 | 0.08196 | 1.87E-01 |
| rs113282230 | chr17:39927157 | Blood Eosinophil Count | 606 | A | T | 0.132 | -0.1084 | 0.08196 | 1.87E-01 |
| rs113571956 | chr17:39927234 | Blood Eosinophil Count | 606 | A | T | 0.132 | -0.1084 | 0.08196 | 1.87E-01 |
| rs73985230 | chr17:39929476 | Blood Eosinophil Count | 606 | T | C | 0.125 | -0.1078 | 0.08357 | 1.97E-01 |
| rs150276395 | chr17:39908449 | Blood Neutrophil Count | 605 | G | A | 0.149 | -0.2002 | 0.08332 | 1.66E-02 |
| rs8065520 | chr17:39915395 | Blood Neutrophil Count | 606 | T | C | 0.269 | -0.06023 | 0.06837 | 3.79E-01 |
| rs73985226 | chr17:39920081 | Blood Neutrophil Count | 606 | A | G | 0.125 | -0.2108 | 0.08864 | 1.77E-02 |
| rs73985227 | chr17:39921437 | Blood Neutrophil Count | 606 | G | A | 0.131 | -0.2207 | 0.08683 | 1.13E-02 |
| rs28623237 | chr17:39924694 | Blood Neutrophil Count | 606 | T | C | 0.194 | -0.1321 | 0.07455 | 7.68E-02 |
| rs73985229 | chr17:39925400 | Blood Neutrophil Count | 606 | G | A | 0.132 | -0.208 | 0.08612 | 1.60E-02 |
| rs113282230 | chr17:39927157 | Blood Neutrophil Count | 606 | A | T | 0.132 | -0.208 | 0.08612 | 1.60E-02 |
| rs113571956 | chr17:39927234 | Blood Neutrophil Count | 606 | A | T | 0.132 | -0.208 | 0.08612 | 1.60E-02 |
| rs73985230 | chr17:39929476 | Blood Neutrophil Count | 606 | T | C | 0.125 | -0.1978 | 0.08788 | 2.48E-02 |

**Table S10.** Numbers (frequency) of African American adult asthmatics by severity in rs2305480-G allele carriers stratified by presence or absence of high-risk haplotype 4. No participants were homozygous for haplotype 4. See also **Fig. 5C**.

| **# rs2305480_G alleles** | **≥1** | **≥1** | **Sample Size** |
| --- | --- | --- | --- |
| **# copies of haplotype 4** | **0** | **1** |  |
| Mild | 15  (0.37) | 2  (0.11) | **19** |
| Moderate | 14  (0.34) | 4  (0.22) | **18** |
| Severe | 12  (0.29) | 12  (0.66) | **26** |
| **Total** | **41** | **18** | **63** |

**Supplementary Figures**

**Fig S1. Overview of study design.**

**
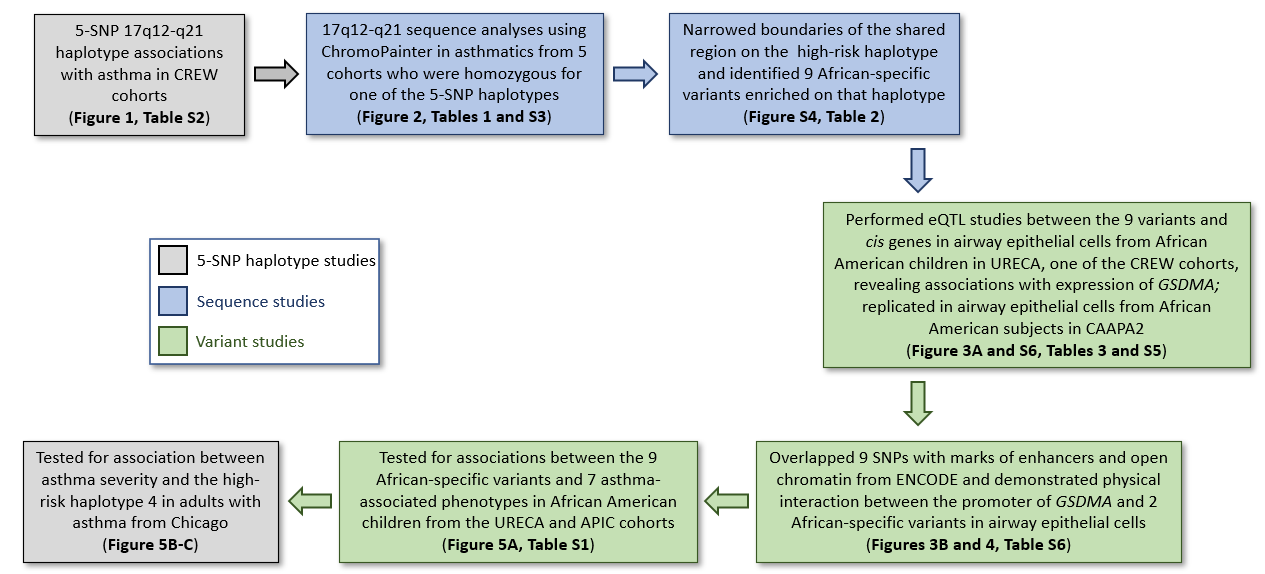
**

**Fig S2. ChromoPainter analysis. Left:** A histogram of the number of chromosomes each index chromosome copied from. **Right:** A histogram of the number of times each index chromosome was copied from.

**
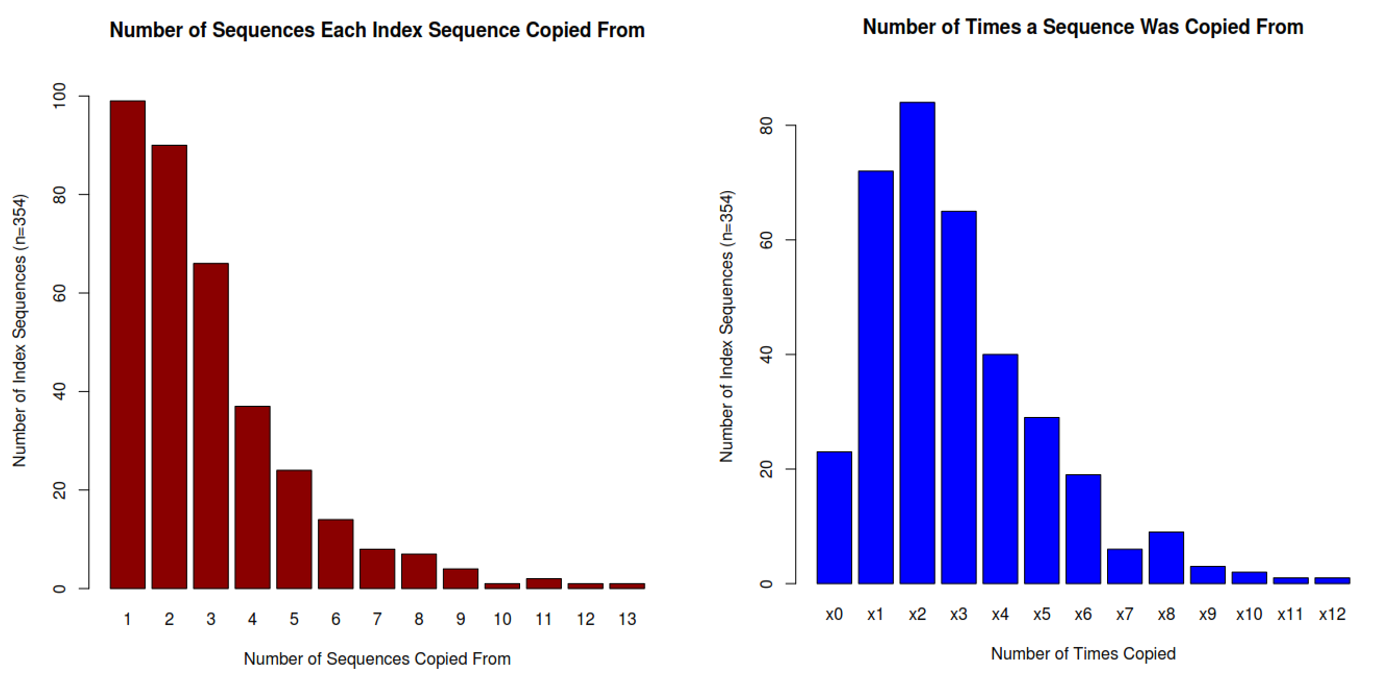
**

**Fig S3. ChromoPainter visualization of haplotype breakpoints at the 17q12-q21 locus in African American sequences.** All 354 African American chromosome samples were randomly divided into four subsets for ease of visualization. The black vertical bars denote the boundaries of the core region.

**
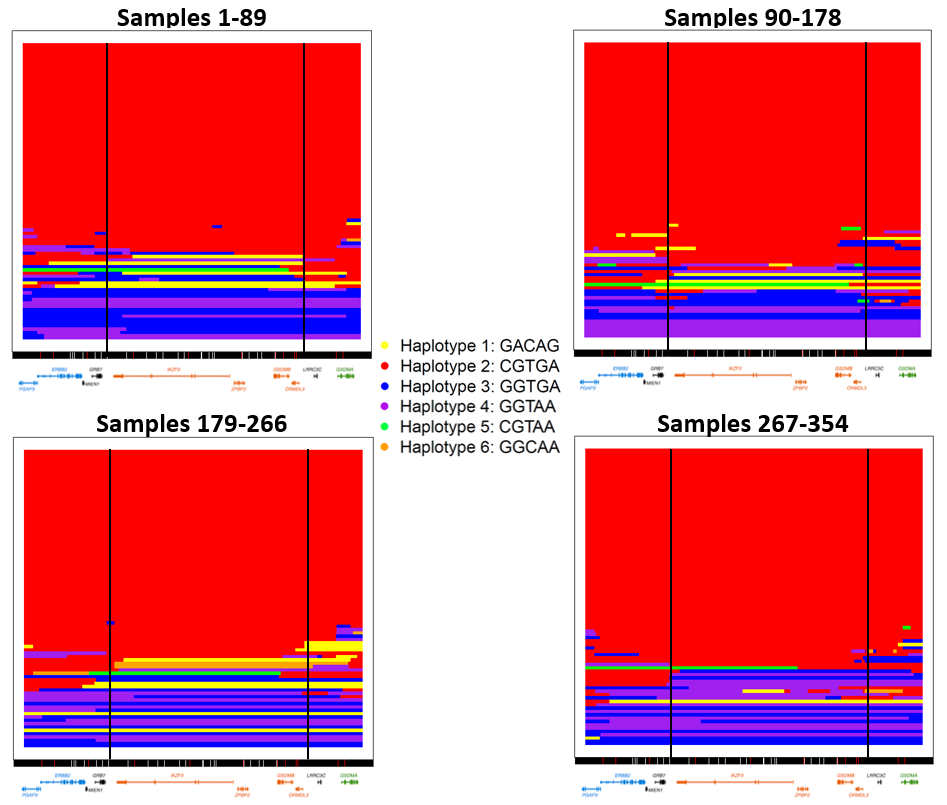
**

**Fig S4**. **ChromoPainter display of the 17q12-q21 core region**. The 36 sequences from asthmatic individuals who were homozygous for the high-risk haplotype 4 are shown. The black box shows the 23.9 kb region shared by all haplotype 4 sequences, and the white vertical lines denote the added ± 1.2 kb to define the 26.3 kb critical region. The four genes and their relative locations in the core region of the locus are shown (chr17:37909547-38100129; build hg19). We required two recombination events to define the shared region and therefore ignored the single recombination event on one chromosome (green bar on the third chromosome from the bottom).


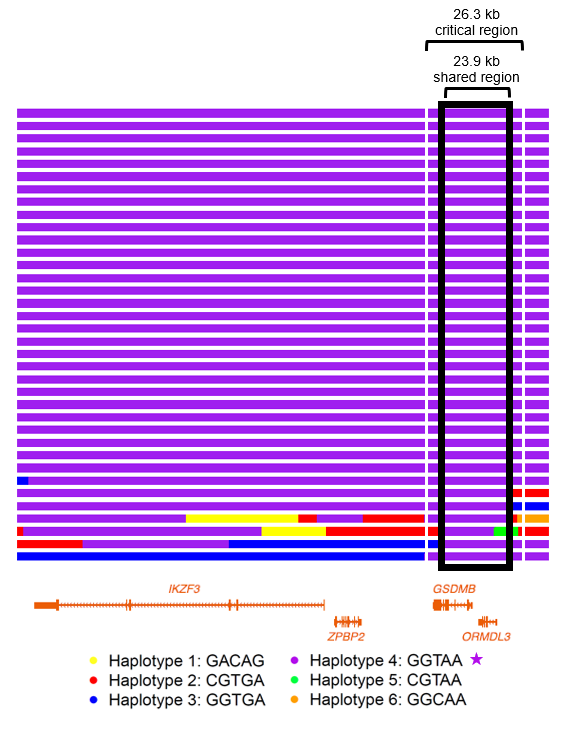


**Fig S5. Ancestry PCA plots for APIC and URECA children.** Ancestry PC1 (x-axis) and PC2 (y-axis) are shown for self-reported Black children in the APIC (left) and URECA (right) cohorts relative to African (YRI), European (CEU), and Asian (CHB/JPT) samples from 1000 Genomes.

**
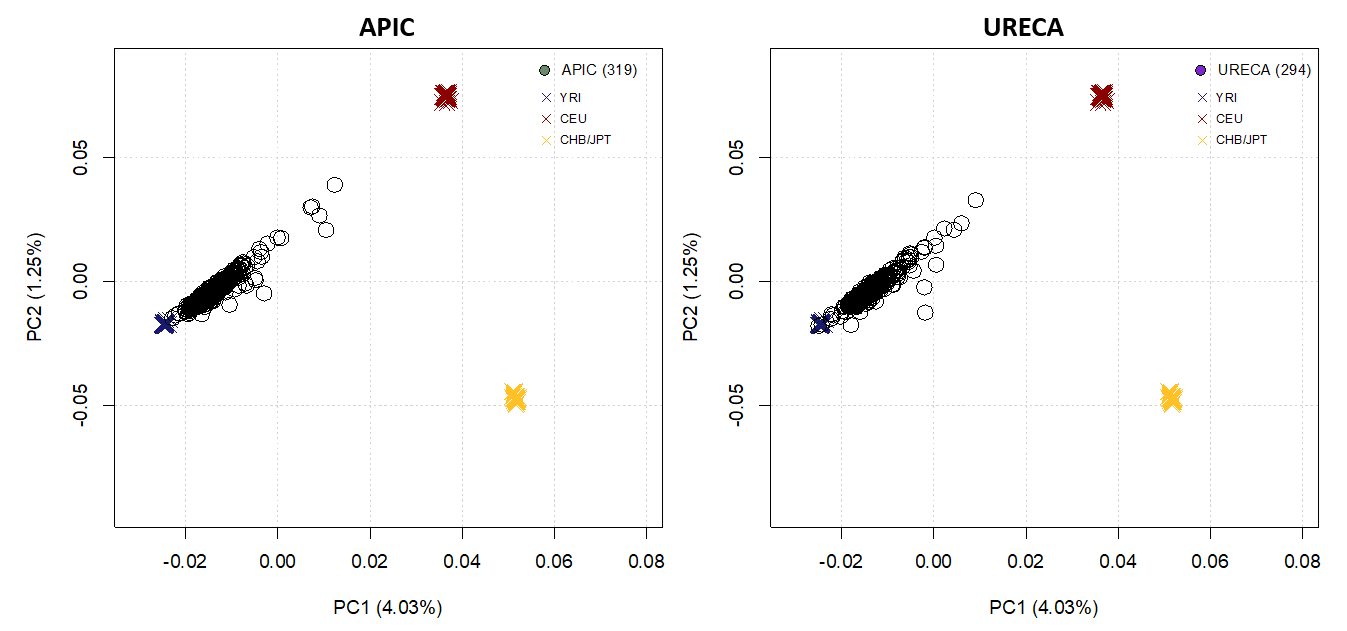
**

**Fig S6. Box plot of rs28623237 eQTL effect on *GSDMA* expression** in nasal epithelial cells from 534 African ancestry subjects from CAAPA2.


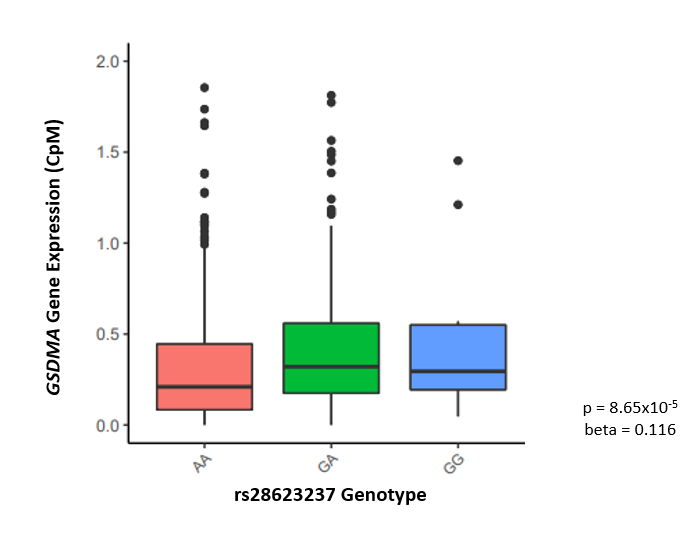


**Fig S7. LD plot of African-specific novel variants and SNPs in or near *GSDMA*.** Red boxes indicate the novel African-specific variants and the blue boxes indicate the most significant eQTLs for SNPs in and near the *GSDMA* gene in airway epithelial cells from 189 self-reported Black children in the URECA cohort (see **Table S4** for eQTL results). Values shown in diamonds are *r*^2^ (black diamonds are *r*^2^ = 1.0) LD data are from 1000G ASW population; region spans chr17:39933720-39978636 (hg38).

**
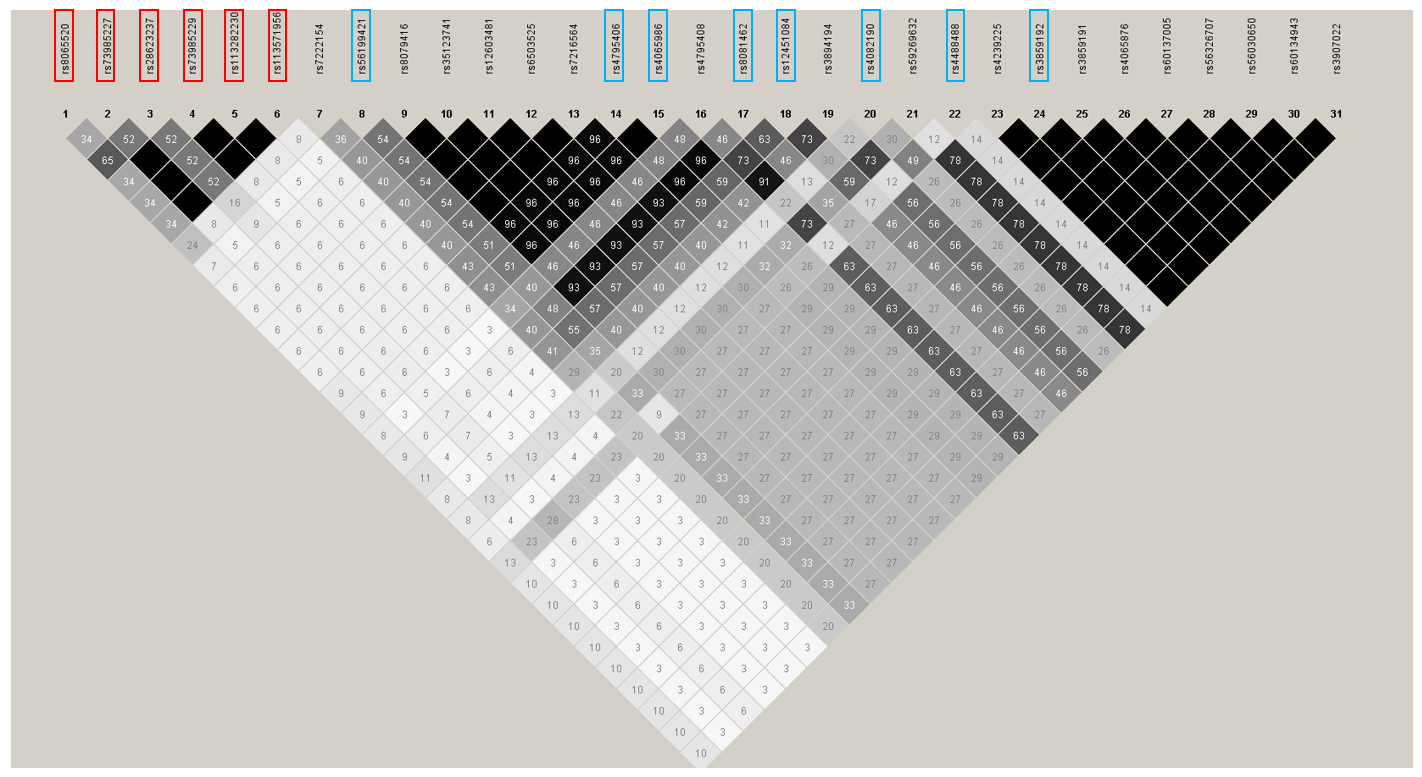
**

**Fig S8. Box plots of rs113282230 eQTL effects on *GSDMA* expression conditioned on SNPs in or near *GSDMA*** in airway epithelial cells from 189 self-reported Black children in the URECA cohort**.** X-axis shows the eQTL effects of rs113282230 on *GSDMA* (left), conditioned on rs3859192 genotype (center), and conditioned on rs4795406 genotype (right). Boxes are colored according to rs113282230 genotype as shown in the legend.

**
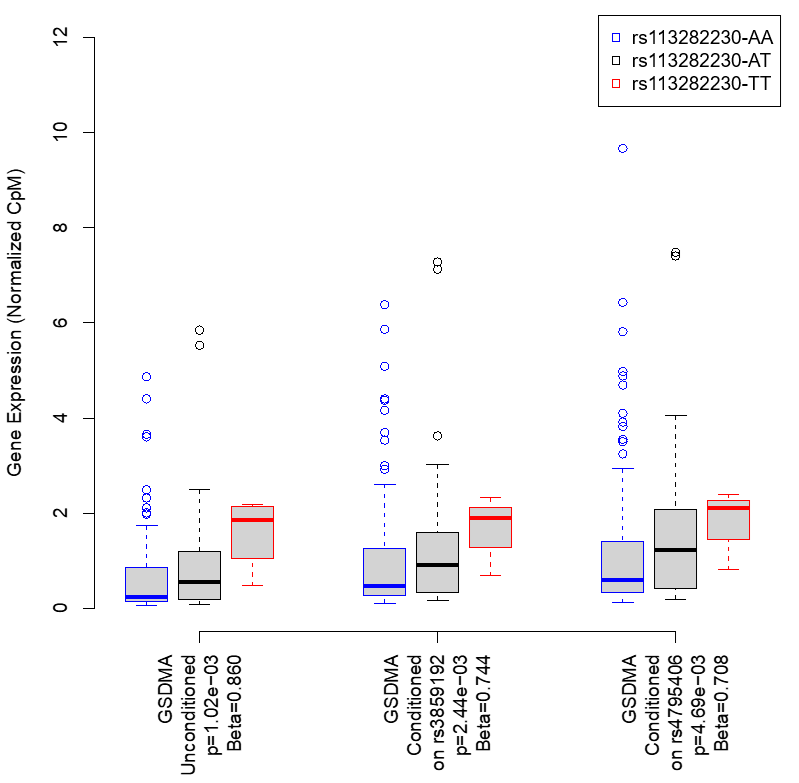
**

**Fig S9. Violin plots of rs2305480 and rs113282230 eQTLs on *GSDMA* and *GSDMB* in airway epithelial cells** in airway epithelial cells from 189 self-reported Black children in the URECA cohort**.** Effects of both SNPs on expression of *GSDMA* (left) and *GSDMB* (right).

**
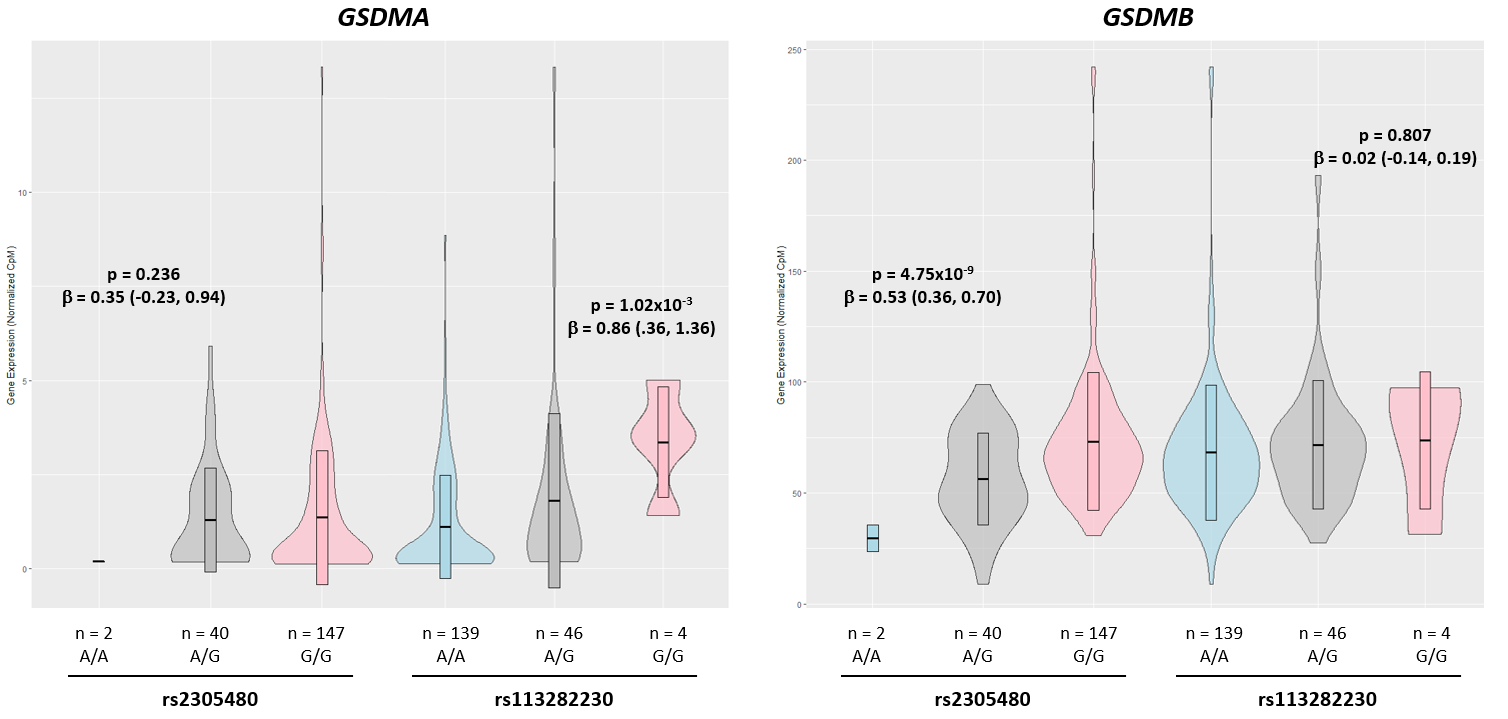
**

**Fig S10. LD plot of the African-specific novel variants and SNPs in the core region of the 17q12-q21 locus.** Linkage disequilibrium plot of the 9 African-specific novel variants (red boxes) and reference SNPs in the core region of the 17q12-21 locus (from Stein *et* al.^20^). Values shown in the diamonds are *r*^2^ (black diamonds are *r*^2^ = 1.0) Data are from 1000G ASW population; region spans chr17:39766006-39929476 (hg38).

**
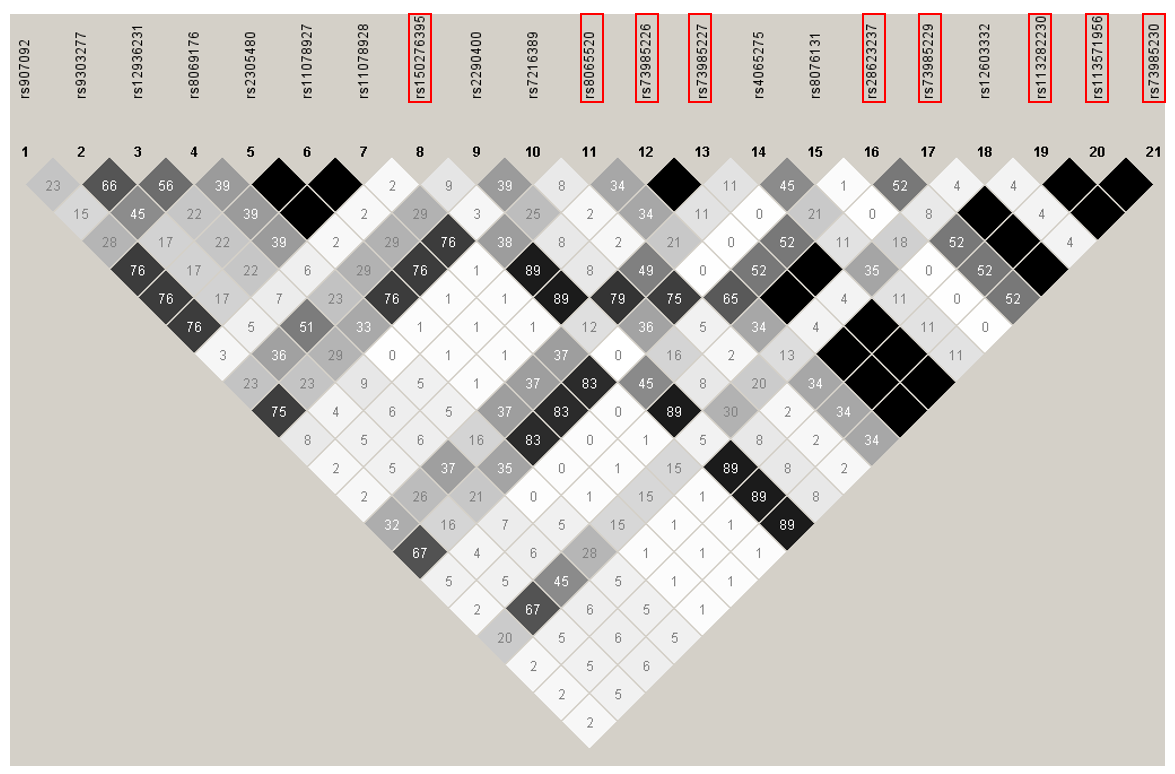
**

**Fig S11. Chromatin annotations in the region encoding the African-specific SNPs in ENCODE cell lines.** From top to bottom: The 3’ end of *GSDMB* and all of *ORMDL3* are shown at the top of the figure, H3K27Ac tracks for NHEK (skin) cells are shown in purple. DNAse hypersensitivity sites in 125 encode cell lines are shown as rectangular boxes; the gray shading corresponds to regions of hypersensitivity (darkness is proportional to the maximum observed signal strength). The number of cell types with open chromatin are shown next to the boxes. DNase hypersensitivity sites for 6 immune cell types are shown in the lower panel. The nine variants are shown at the bottom of the figure; the two variants in the candidate region are designated by an orange arrow and box and tracked vertically by a dashed orange line.

**
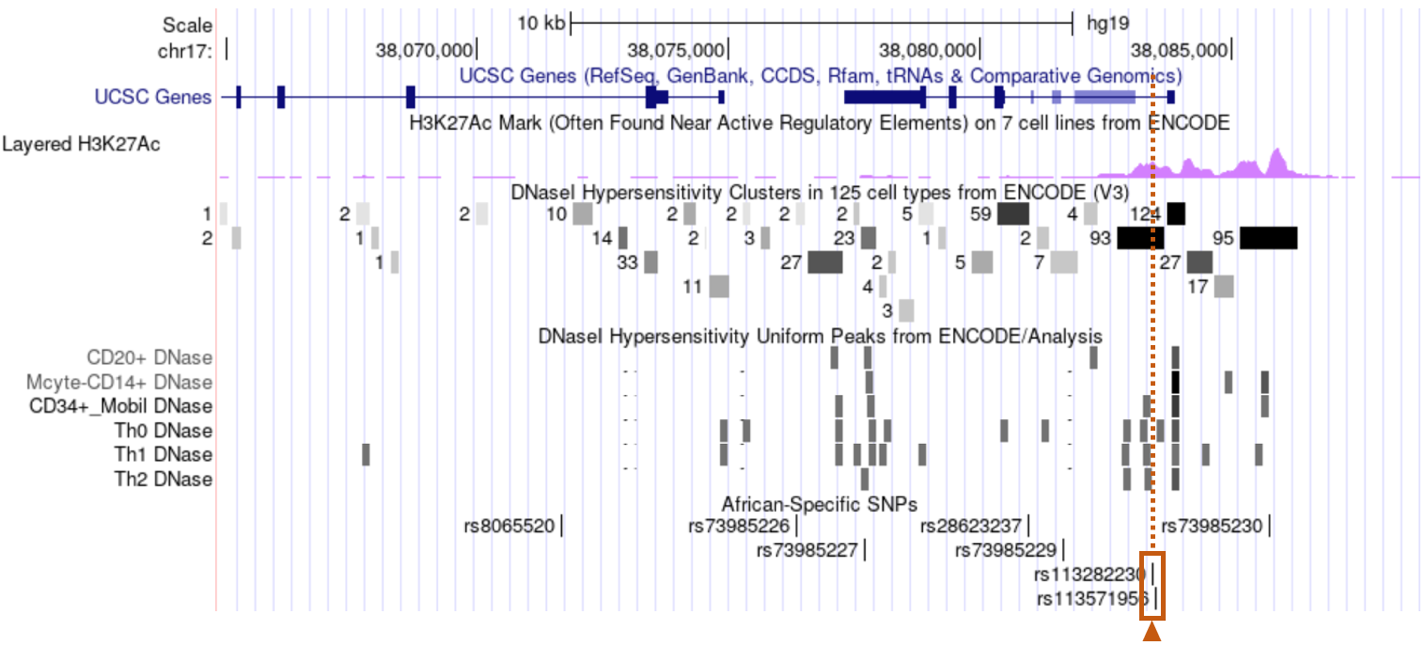
**

**Fig S12. eGenes for rs113282230 at the 17q12-q21 locus in immune cells** (from the eQTL Browser: <https://fivex.sph.umich.edu/variant/eqtl/17_39927157?group_by=symbol&n_labels=5&study%5B%5D=Schmiedel_2018&tss_distance=500000&y_field=log_pvalue>). All eQTLs within a ±500 kb window are shown in the DICE study^21^. The frequency of rs113282230-T was 0.011 in this sample.

**
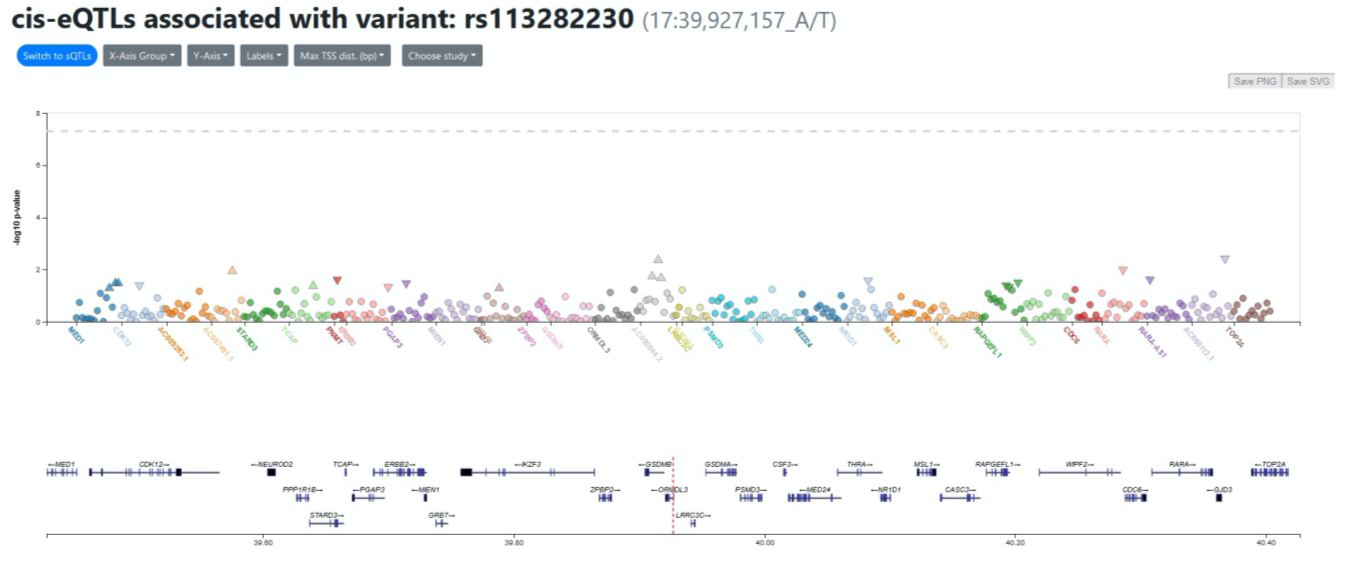
**

**Fig S13. pcHi-C data for rs113282230 in immune cells.** pcHi-C interactions with the nine African-specific variants in immune cells from Javier et al.^22^ as displayed in the Open Target browser: (<https://genetics.opentargets.org/variant/17_39927157_A_T>). Aggregated scores represent the position in the quantile distribution of interaction scores across immune cell types (1 is the strongest interaction). n.i, variant not included; blank cell, no interaction. See also **Additional File 1: Table S8**).

**
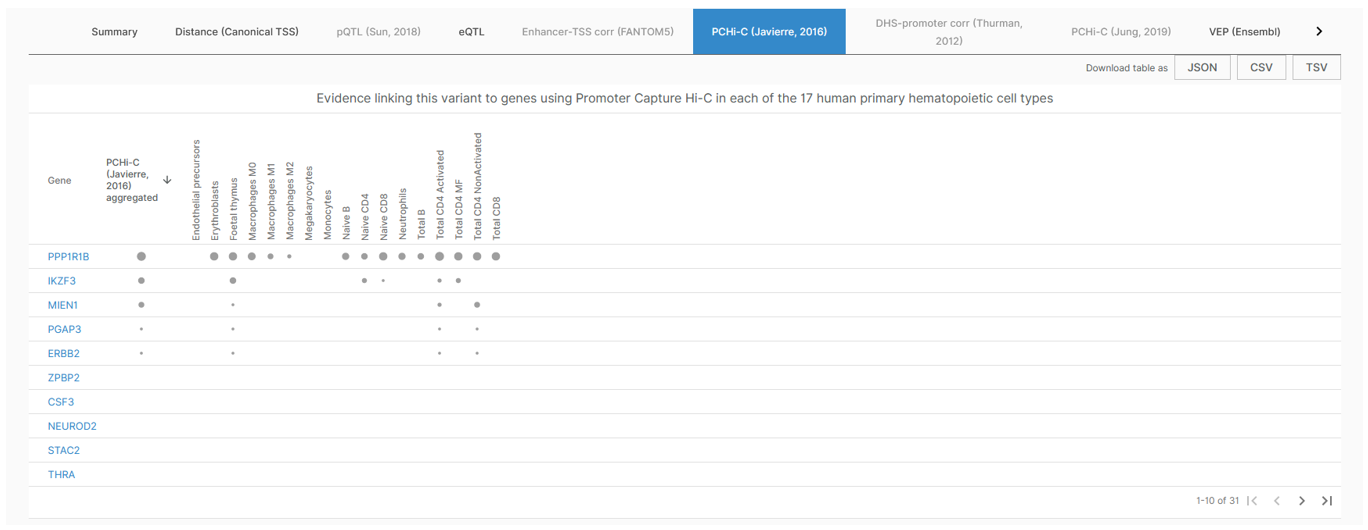
**

**Fig S14. rs113282230 genotype effect on asthma prevalence by rs2305480 AA and GG genotype.** Data from 371 APIC and URECA cases and 248 URECA and CAAPA controls. The lowest risk group (0 risk alleles for both genotypes) shown in yellow. Among the rs2308480 GG homozygotes, increasing copies of the rs113282230 risk allele is shown with darker grey coloring. Error bars show 2 standard deviations.

**
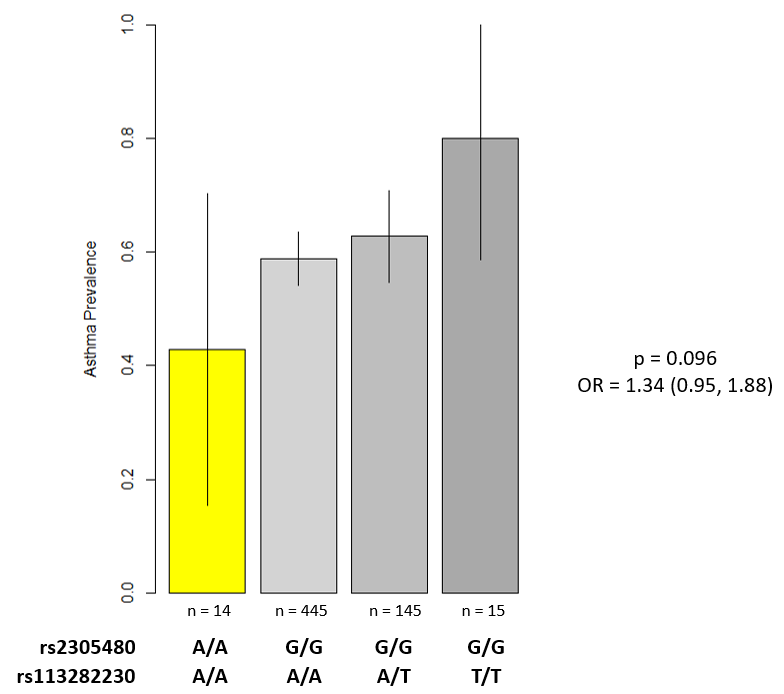
**

**References**

1. Gern, J. E. *et al.* The Children's Respiratory and Environmental Workgroup (CREW) birth cohort consortium: design, methods, and study population. *Respir Res* **20**, 115, doi:10.1186/s12931-019-1088-9 (2019).

2. Ober, C. *et al.* Expression quantitative trait locus fine mapping of the 17q12-21 asthma locus in African American children: a genetic association and gene expression study. *Lancet Respir Med* **8**, 482-492, doi:10.1016/S2213-2600(20)30011-4 (2020).

3. Igartua, C. *et al.* Ethnic-specific associations of rare and low-frequency DNA sequence variants with asthma. *Nat Commun* **6**, 5965, doi:10.1038/ncomms6965 (2015).

4. Torgerson, D. G. *et al.* Meta-analysis of genome-wide association studies of asthma in ethnically diverse North American populations. *Nat Genet* **43**, 887-892, doi:10.1038/ng.888 (2011).

5. Levin, A. M. *et al.* A meta-analysis of genome-wide association studies for serum total IgE in diverse study populations. *J Allergy Clin Immunol* **131**, 1176-1184, doi:10.1016/j.jaci.2012.10.002 (2013).

6. Myers, R. A. *et al.* Further replication studies of the EVE Consortium meta-analysis identifies 2 asthma risk loci in European Americans. *J Allergy Clin Immunol* **130**, 1294-1301, doi:10.1016/j.jaci.2012.07.054 (2012).

7. Myers, R. A. *et al.* Genome-wide interaction studies reveal sex-specific asthma risk alleles. *Human molecular genetics* **23**, 5251-5259, doi:10.1093/hmg/ddu222 (2014).

8. Dapas, M. *et al.* Multi-omic association study implicates *PPP1R13B* in DNA methylation-mediated genotype and smoking exposure effects on decreased lung function in urban children. *medRxiv*, doi:<https://doi.org/10.1101/2022.06.24.22276830> (2022).

9. Zoratti, E. M. *et al.* Asthma phenotypes in inner-city children. *J Allergy Clin Immunol* **138**, 1016-1029, doi:10.1016/j.jaci.2016.06.061 (2016).

10. Gergen, P. J., Teach, S. J., Togias, A. & Busse, W. W. Reducing Exacerbations in the Inner City: Lessons from the Inner-City Asthma Consortium (ICAC). *J Allergy Clin Immunol Pract* **4**, 22-26, doi:10.1016/j.jaip.2015.07.024 (2016).

11. Gern, J. E. *et al.* The Urban Environment and Childhood Asthma (URECA) birth cohort study: design, methods, and study population. *BMC Pulm Med* **9**, 17 (2009).

12. Altman, M. C. *et al.* Endotype of allergic asthma with airway obstruction in urban children. *J Allergy Clin Immunol*, doi:10.1016/j.jaci.2021.02.040 (2021).

13. O'Connor, G. T. *et al.* Early-life home environment and risk of asthma among inner-city children. *J Allergy Clin Immunol* **141**, 1468-1475, doi:10.1016/j.jaci.2017.06.040 (2018).

14. Magnaye, K. M. *et al.* A-to-I editing of miR-200b-3p in airway cells is associated with moderate-to-severe asthma. *Eur Respir J* **58**, doi:10.1183/13993003.03862-2020 (2021).

15. Nicodemus-Johnson, J. *et al.* DNA methylation in lung cells is associated with asthma endotypes and genetic risk. *JCI Insight* **1**, e90151, doi:10.1172/jci.insight.90151 (2016).

16. Expert Panel Report 3 (EPR-3): Guidelines for the Diagnosis and Management of Asthma-Summary Report 2007. *J Allergy Clin Immunol* **120**, S94-138 (2007).

17. Cornish-Bowden, A. Nomenclature for incompletely specified bases in nucleic acid sequences: recommendations 1984. *Nucleic Acids Res* **13**, 3021-3030, doi:10.1093/nar/13.9.3021 (1985).

18. Madeira, F. *et al.* The EMBL-EBI search and sequence analysis tools APIs in 2019. *Nucleic Acids Res* **47**, W636-W641, doi:10.1093/nar/gkz268 (2019).

19. Schaid, D. J., Rowland, C. M., Tines, D. E., Jacobson, R. M. & Poland, G. A. Score tests for association between traits and haplotypes when linkage phase is ambiguous. *Am J Hum Genet* **70**, 425-434, doi:10.1086/338688 (2002).

20. Stein, M. M. *et al.* A decade of research on the 17q12-21 asthma locus: Piecing together the puzzle. *J Allergy Clin Immunol* **142**, 749-764 e743, doi:10.1016/j.jaci.2017.12.974 (2018).

21. Schmiedel, B. J. *et al.* Impact of Genetic Polymorphisms on Human Immune Cell Gene Expression. *Cell* **175**, 1701-1715 e1716, doi:10.1016/j.cell.2018.10.022 (2018).

22. Javierre, B. M. *et al.* Lineage-Specific Genome Architecture Links Enhancers and Non-coding Disease Variants to Target Gene Promoters. *Cell* **167**, 1369-1384 e1319, doi:10.1016/j.cell.2016.09.037 (2016).
